# Initiating the commercialization of genetically modified staple crops in China: domestic biotechnological advancements, regulatory milestones, and governance frameworks

**DOI:** 10.1080/21645698.2025.2520664

**Published:** 2025-06-20

**Authors:** Tianheng Mu, Qiuchi Song, Yonghong Liu, Jun Song

**Affiliations:** a Department of Sociology, University of Warwick Sociology, Coventry, UK; b Department of Cell Biology and Molecular Genetics, University of Maryland, College Park, MD USA; c Institute of Agricultural Quality Standards and Testing Technology, Sichuan Academy of Agricultural Science, Chengdu, PR China; d Chengdu Center for Food Quality Supervision, Inspection and Testing, Ministry of Agriculture and Rural Affairs, Chengdu, PR China

**Keywords:** Challenges and solutions, Chinese governance model, commercialization of GM technology, regulatory evolution

## Abstract

Historically, China’s approach to genetically modified (GM) staple crops has been cautious. However, in 2021, China launched its first pilot program for the commercial cultivation of GM food crops and subsequently expanded their cultivation. Previously, the only GM plants cultivated in China were insect-resistant cotton and virus-resistant papaya. The regulatory and policy shifts, from initial research and cautious development to accelerated commercialization, led to significant changes in China’s safety-related regulatory framework for genetically modified organism (GMOs). Here, China’s progress in GMO research and commercialization over the past three decades is comprehensively analyzed. This review traces the evolution of core regulations governing GMO safety, summarizes the Chinese model of GMO safety governance, and highlights the remaining challenges as commercialization progresses. The goal was to present the international community with a GMO safety governance model reflecting China’s characteristics and practices, offering a Chinese solution to balancing GMO commercial adoption with ecological preservation.

## 1. Introduction

Population growth presents significant challenges to agricultural production, with the most pressing issue being the need to address hunger and poverty.^1^ An estimated one billion people worldwide experience varying degrees of hunger, with approximately 870 million suffering from malnutrition. The majority of those affected reside in developing countries in Africa, Asia, and South America.^2^ Climate change, environmental degradation, and the diminishing amount of arable land exacerbate the challenges of satisfying the increasing global food demand.^3^ Over the past three decades, advances in biotechnology, particularly genetically modified (GM) food technologies, have offered effective tools for increasing food production, improving food quality, and mitigating environmental degradation.^4,5^ In the new wave of agricultural advancements, disruptive GM technologies and their products have the potential to address many issues related to world hunger and malnutrition.^6–8^ GM crops have already been widely recognized for their ability to address agricultural problems, such as yield losses due to pests, weed proliferation, and drought. In 2023, the global planting area of GM food crops reached 206.3 million hectares, an increase of 121× since 1996, representing approximately 13.38% of the total global arable land. The cumulative planting area exceeded 3.4 billion hectares.^9^ China was among the earliest countries globally to initiate the commercial cultivation of GM plants. However, prior to 2021, commercialization efforts were largely limited to two economic plants: insect-resistant cotton and virus-resistant papaya. These applications had not yet extended to staple food crops. According to the official definition provided by the Ministry of Agriculture and Rural Affairs (MARA), staple food crops refer to those that play a fundamental role in the national dietary structure, are characterized by large-scale cultivation and high yield, and primarily serve as sources of carbohydrates. Traditionally, this category includes rice, wheat, maize, and potato. Notably, with the evolution of China’s modern food security strategy, the concept of staple crops has expanded beyond ensuring basic production levels. It now encompasses broader dimensions such as nutritional adequacy, resilience of the entire agri-food supply chain, and long-term ecological sustainability. Given the sustained high dependence on soybean imports and the associated strategic risks, China has officially classified soybean as a staple crop and prioritized it as a key focus area in its national food security agenda. In 2021, China initiated small-scale trials of domestically developed GM corn and soybean at research bases, achieving the expected outcomes in yield improvement, pest resistance, and weed control. Consequently, China expanded the cultivation of GM corn and soybean from 2022 to 2024. By 2024, GM corn and soybean were planted across more than 20 counties in 8 provinces, covering over 1.7 million acres.^10^ However, during the development and commercialization of GM technology and GM food crops, public concerns regarding the potential risks of GM foods to human health and the environment have persisted.^11,12^ Despite more than three decades of widespread commercial cumulative cultivation and consumption of GM crops, spanning nearly 4 billion hectares globally and involving billions of meals containing GM ingredients, public concerns over their potential risks to human health and the environment have persisted.^11,12^ Extensive scientific evidence accumulated over this period has not produced any credible indication of GM foods having adverse effects on human or animal health, or on ecological systems. The longstanding cultivation of GM crops in countries such as the United States, the import and processing of GM food products in nations that do not themselves grow GM crops, and the routine consumption of GM foods by international visitors further support the conclusion that GM foods are safe for human consumption and do not pose environmental hazards. A wealth of literature and data indicate that GM food crops do not significantly differ from non-GM crops in their effects on soil microorganisms, plants, non-target animals, and human and animal health.^11^ Thus, both theoretical and practical evidence suggests that there are no substantial effects of GM and non-GM foods on health and the environment. Despite the established safety of GM foods and the rigorous safety assessments conducted by government regulatory agencies for each new GM crop prior to commercialization,^13,14^ debates about the safety of GM crops and their derived foods and feeds continue. These debates often overlook a crucial fact: all the raw materials used for human food or animal feed have been derived from crops produced through conventional crossbreeding. During the crossbreeding process, large-scale genomic changes occur,^15^ but they are not subject to molecular identification. In contrast to traditional breeding, GM food crops are developed using precisely defined and well-understood genes, with the resulting biotechnological products undergoing rigorous testing and evaluation to address any potential environmental or health safety concerns prior to their release.

Food safety is a shared concern among scientists, policymakers, and consumers, particularly as foodborne illnesses become more frequent and GM foods receive increasing scrutiny. As a result, governments worldwide have made concerted efforts to regulate the production and trade of GM foods. Research into the safety of GM foods, conducted by both regulatory bodies and the scientific community, has remained constant, with significant resources allocated by research institutions, universities, and international organizations for in-depth studies. These studies address various aspects, including the nutritional composition, toxicity, allergenicity, antibiotic resistance, gene flow, and impact on the biodiversity of GM crops and their products. Although existing evidence indicates that the safety of GM foods is comparable to that of conventional foods, concerns persist within the scientific community and regulatory agencies regarding the long-term effects and potential risks of GM technology, thereby necessitating comprehensive safety assessments. In response, countries have developed governance systems for genetically modified organisms (GMOs) that regulate safety assessments, labeling, testing, and approval processes. The establishment and refinement of these systems have helped standardize GM technology-related research and applications, ensuring the legality and safety of GM foods on the market. Simultaneously, governments have strengthened oversight, employing regular testing and risk assessments, to ensure the effective management of GM food safety. Although there is a broad international consensus on the need to enhance the regulation and evaluation of GM technologies to ensure their safety, significant differences persist across countries and regions in GM food-related safety evaluations and administrative frameworks.^16^

The formulation and evolution of regulations and policies for GM technologies reflect a country’s or region’s stance on the commercial application of GMOs. China’s development of GMO safety regulations and governance systems began later than those of the United States and the European Union (EU).^17^ However, over more than three decades, China has developed a comprehensive, nation-specific GMO safety governance framework. This system safeguards human health and environmental ecology while promoting the sustainable development of the agricultural biotechnology sector. Over the past 30 years, China has revised its agricultural GMO safety governance regulations and policies several times, reducing barriers to commercial releases and facilitating a shift from cautious development to accelerated GMO commercialization. As a result, 2023 has been referred to as the “initial year” of China’s GM staple crop commercialization, signaling the entry of this process into a new, fast-paced phase. Here, the key achievements in China’s GMO research over the past three decades are examined, the progress in commercializing these innovations is evaluated, and the changes in China’s core GMO regulatory framework are explored. Additionally, the characteristics of China’s GMO safety governance model are identified. A comprehensive and systematic analysis of China’s agricultural biotechnology advancements, as well as their commercial applications, is provided. The evolution of the regulatory system, focusing on the *Regulation on the Safety Administration of Agricultural GMOs* and *Administrative Rule for Safety Evaluation of Agricultural GMOs*, is traced, and the Chinese model of GMO safety governance is outlined. Furthermore, the challenges that must be overcome in the ongoing GM crop commercialization process are addressed. This study is the first to thoroughly examine the evolution of China’s GMO safety governance regulations and policies in the context of the official commencement of GMO staple food crop commercialization. The objective was to offer insights into how China aims to balance GMO commercialization with ecological and environmental protection.

## 2. Evolution of Agricultural GMO Safety Governance Regulations and Policies

China’s regulatory framework for the safety governance of GMOs dates back to the 1990s. In 1993, the Ministry of Science and Technology (MST) announced the first administrative rule for GMO safety, the *Administrative Rule for Genetic Engineering Safety*, aimed at promoting biotechnology research, enhancing safety governance in genetic engineering, and safeguarding public and researcher health. This rule designated MST as the authority overseeing national genetic engineering safety, and the National Genetic Engineering Safety Committee was established to supervise and coordinate efforts. Various State Council (SC) administrative bodies were assigned specific safety governance responsibilities within their respective domains. However, after a period of implementation, the rule proved ineffective and became largely inactive. Subsequently, the MARA and the National Health Commission issued the *Administrative Rule for Agricultural Genetic Engineering Safety* and the *Rule for the Hygienic Administration of GM Foods*, respectively. However, these rules failed to keep pace with the growing regulatory needs of the rapidly advancing GM technology and were eventually repealed. In 2001, the SC enacted the *Regulation on the Safety Administration of Agricultural GMOs*, which introduced detailed provisions on safety evaluation, labeling, production, and business licensing, as well as import safety approval systems, for agricultural GMOs. This regulation represents China’s first and, to date, most of the significant legislation has specifically focused on the safety governance of agricultural GMOs, and it remains the cornerstone regulation for governing GM food safety at its source. Following the issuance of this legislation, the MARA released several related rules, including the *Administrative Rule for Safety Evaluation of Agricultural GMOs*, the *Administrative Rule for the Safety of Imported Agricultural GMOs*, the *Rule on the Approval of Processing of Agricultural GMOs*, and the *Administrative Rule on the Labeling of Agricultural GMOs*. Additionally, the State Administration for Market Regulation issued the *Rule on the Inspection and Quarantine of Imported and Exported GMOs* (Table 1). These regulations and rules have undergone multiple revisions and remain in effect today. Thus, China has developed a regulatory framework based on the SC’s regulations and MARA’s implementation rules (core regulations and rules) (Table 1). In addition, other relevant laws, such as the *Seed Law*, the *Agricultural Product Quality Safety Law*, the *Food Safety Law*, the *Biosafety Law*, and the *Rule on Safety Review of New Food Materials*, include provisions on GM food safety. However, these provisions are primarily derived from the core regulations and rules mentioned above, with some directly citing specific provisions. China’s core regulatory framework for GMO safety has established a phased and progressive approval system that governs the development of GMOs from laboratory research through intermediate trials, environmental release, and productive field trials. This system is grounded in a risk-based management logic that emphasizes the stepwise validation of biosafety and ecological risks at different stages and scales of experimentation. During the laboratory research phase, activities such as gene cloning, vector construction, and gene editing must be conducted in physically contained laboratories, with strict prohibition against any contact with the natural environment. Developers are required to submit experimental protocols to the regulatory authority, namely, the MARA, for record-keeping. The MARA then evaluates the biosafety levels of the donor and recipient organisms and verifies laboratory qualifications. The intermediate trial phase involves the first field-level validation, conducted in enclosed plots of less than 0.165 acres, such as greenhouses or net houses, with the objective of assessing trait expression stability. Developers must submit a biosafety assessment application, which is reviewed by expert panels under the National Biosafety Committee. Particular attention is given to the adequacy of physical containment and emergency response measures. The environmental release phase allows for semi-open field testing in plots smaller than 0.82 acres, in which limited pollen dispersal is permitted, but a 500-meter isolation buffer is required. Developers must provide data from intermediate trials along with an environmental monitoring plan. A new round of expert review is then conducted, focusing on the continued effectiveness of containment and emergency protocols. The productive trial phase, which was introduced on the basis of China’s principle of incremental risk management, involves large-scale agronomic testing, exceeding 0.82 acres, at designated trial sites, following MARA approval. These trials span 2–3 years and are designed to collect comprehensive data on yield, stress resistance, and ecological impacts on non-target organisms under near-commercial conditions. This stage, introduced under the 2006 National Medium- and Long-Term Science and Technology Development Plan, aims to evaluate gene flow control effectiveness and assess potential biosafety implications of actual farming operations, including mechanical harvesting. It also helps establish baseline biodiversity data under scaled-up conditions. Supervision during the productive trial phase has been significantly strengthened. Key regulatory measures include the implementation of a “dual-lock” system requiring both electronic fencing (to record personnel access) and physical barriers (e.g., 8-meter-wide non-GM buffer crop zones). Field management data must be recorded using blockchain technology and uploaded in real time to the national GMO biosafety information platform. Independent ecological impact assessments must be conducted by third-party institutions, typically national research institutes. China’s uniquely designed productive trial phase exemplifies the institutional application of the precautionary principle. It responds to two distinctive national circumstances: (1) the vulnerability of its smallholder farming system, which comprises roughly 230 million small farms, many of which lack the adequate capacity to manage biotech-related risks; and (2) the country’s status as one of 12 global centers of crop origin, which necessitates stringent safeguards against gene flow to wild relatives. Although this four-tiered regulatory framework increases the time and cost associated with GMO development, it effectively establishes a globally distinctive biosafety buffer. By conducting area-restricted, stage-specific trials, China has adopted a cautious yet forward-moving approach that mitigates ecological risks while building a robust system for biosafety validation ahead of the full-scale commercialization of GM food crops. Productive trials of a certain scale in China balance stringent biosafety oversight with the efficiency demands of scientific and technological development. In recent years, particularly 2021 and 2022, significant revisions were made to these core regulations and rules to facilitate the commercialization of GM food crops and mitigate regulatory barriers to their commercialization.

**Table 1.** Overview of China’s GMO safety administration laws, regulations, rules, provisions, and standards.

No.	Laws, Regulations, Rules, Provisions, and Standards	Institutions Responsible for Promulgating	Revisions	Status
1	Regulation on the Safety Administration of Agricultural GMOs	State Council, 2001	Revised in 2011 and 2017	Valid
2	Administrative Rule for Safety Evaluation of Agricultural GMOs	Ministry of Agriculture and Rural Affairs, 2002	Revised in 2004, 2016, and 2022	Valid
3	Administrative Rule for the Safety of Imported Agricultural GMOs	Ministry of Agriculture and Rural Affairs, 2002	Revised in 2004 and 2017	Valid
4	Rule on the Approval of Processing of Agricultural GMOs	Ministry of Agriculture and Rural Affairs, 2002	No revisions	Valid
5	Administrative Rule on the Labeling of Agricultural GMOs	Ministry of Agriculture and Rural Affairs, 2002	Under revision	Under revision
6	Rule on the Inspection and Quarantine of Imported and Exported GMOs	State Administration for Market Regulation, 2004	Revised in March, April, and November 2018	Valid
7	Rule on the Approval of Major Crop Varieties	Ministry of Agriculture and Rural Affairs, 2016	Revised in March 2019 and 2022	Valid
8	Provisions on the Naming of Agricultural Plant Varieties	Ministry of Agriculture and Rural Affairs, 2012	Revised in 2022	Valid
9	Administrative Rule on Seed Production and Sales Licenses	Ministry of Agriculture and Rural Affairs, 2016	Revised in 2017, 2019, 2020, and 2022	Valid
10	Technical Standards for the Approval of GM Maize Varieties	Ministry of Agriculture and Rural Affairs, 2022	No revisions	Valid
11	Technical Standards for the Approval of GM Soybean Varieties	Ministry of Agriculture and Rural Affairs, 2022	No revisions	Valid
12	Guidelines for the Safety Evaluation of Genetically Modified Plants	Ministry of Agriculture and Rural Affairs, 2017	Revised in 2022	Valid
13	Guidelines for the Safety Evaluation of Agricultural Gene-Edited Plants	Ministry of Agriculture and Rural Affairs, 2022	No revisions	Valid
14	Provisions on the Examination of Agricultural Gene-Edited Plants	Ministry of Agriculture and Rural Affairs, 2023	No revisions	Valid
15	Seed Law of the People’s Republic of China	National People’s Congress, 2000	Revised in 2004, 2013, 2015, and 2022	Valid
16	Law on the Quality and Safety of Agricultural Products	National People’s Congress, 2006	Revised in 2018 and 2022	Valid
17	Food Safety Law of the People’s Republic of China	National People’s Congress, 2009	Revised in 2015, 2018, and 2021	Valid
18	Biosafety Law of the People’s Republic of China	National People’s Congress, 2020	Revised in 2024	Valid
19	Administrative Rule for the Examination of New Food Raw Material Safety	National Health Commission, 2013	Revised in 2017	Valid
20	Hygienic Administrative Rule for New Resource Foods	National Health Commission, 2006	No revisions	Abolished
21	Administrative Rule for Genetic Engineering Safety	Ministry of Science and Technology, 1993	No revisions	Valid
22	Administrative Rule for Agricultural Genetic Engineering Safety	Ministry of Agriculture and Rural Affairs, 1996	No revisions	Abolished
23	Rule for the Hygienic Administration of GM Foods	National Health Commission, 2002	No revisions	Abolished

### 2.1. Regulating the Safety Administration of Agricultural GMOs

The *Regulation on the Safety Administration of Agricultural GMOs*, enacted in 2001, is the highest-level and most authoritative legislation in China’s regulatory framework for agricultural GMOs. Issued directly by the SC, this regulation takes precedence over other related rules issued by various ministries and covers all activities involving GMOs within China. The regulation was revised in 2011 and 2017 to address evolving needs. The 2011 revision introduced provisions for the supervision and governance of GM food safety, designating local governments at, or above, the county level as responsible authorities, in accordance with the *Food Safety Law*. In comparison to the 2011 version, the current regulation specifies that safety testing for GMOs seeking certification must be carried out by accredited institutions designated by the MARA. This change reflects a shift toward greater oversight by the MARA and a reduced reliance on safety reports submitted by applicants. However, this revision also benefits applicants because the government now covers the cost of safety testing, thereby reducing the applicant’s financial burden. The 2017 revision streamlined the process for approving GMOs and products for cross-border movement. Previously, approvals were handled jointly by the MARA and the General Administration of Customs, but under the new regulation, this responsibility was solely assigned to the MARA, further consolidating its regulatory authority.

### 2.2. Administrative Rule for Safety Evaluation of Agricultural GMOs

The *Administrative Rule for Safety Evaluation of Agricultural GMOs*, issued by the MARA in 2002, mandates that all the activities involving agricultural GMOs in China, such as research, testing, production, processing, trade, and import/export, must undergo safety evaluations. This rule has been in effect for 22 years and has undergone four revisions, in 2004, 2016, 2017, and 2022, with the latter being particularly significant in facilitating the commercialization of GM food crops. The rule outlines specific requirements for safety evaluations related to intermediate testing, environmental release, and production trials, as well as the documentation required for safety certificate applications (Tables 2–5). The revisions in 2004, 2016, and 2017 were relatively minor, introducing no substantial changes. However, the 2022 revision introduced several key changes aimed at improving evaluation efficiency and simplifying procedures to support the commercialization of GM food crops. The following four main changes were introduced in 2022: (1) encouraging researchers to establish or share dedicated experimental bases for agricultural GMOs, thereby optimizing the use of limited testing facilities and accelerating the development of GM varieties; (2) modifying terminology in testing plans from “varieties” and “lines” to “transformants” and allowing derivative varieties or lines of certified transformants to bypass reevaluation, thereby reducing evaluation procedures, saving applicant time and costs, and promoting industry growth; (3) altering production trial requirements by allowing them to be conducted in appropriate agricultural ecological zones rather than only in provinces with environmental release approval, thereby streamlining the approval process and reducing applicant time and costs; (4) and revising the safety evaluation requirements for hybrid offspring of transgenic plants, allowing combinations of two or more certified transformants to begin evaluation at the production trial stage, thereby further simplifying the evaluation process. Additionally, the revised rule stipulates that when transgenic plants are used as parents in hybridization with conventional varieties, safety evaluations must begin at the production trial stage. Furthermore, for the commercial production of GM crops, applicants must submit a report from a qualified testing institution confirming the consistency between the GM crop’s target traits and the characteristics of the transformants. The 2022 revisions optimized the approval process, reduced the burden on applicants, and waived certain testing fees, thereby facilitating the commercialization of GM food crops in China. Importantly, these revisions did not reduce the regulatory oversight of GMO safety. In addition, the MARA has updated the *Guidelines for the Safety Evaluation of GM Plants*, developed the *Guidelines for the Safety Evaluation of Agricultural Gene-Edited Plants*, and established the *Provisions on the Examination of Agricultural Gene-Edited Plants* to enhance the operability of agricultural biotechnology safety evaluations.

**Table 2.** Application documentation required for intermediate trial permission under China’s GMO safety governance regulations.

No.	Required application documentation for intermediate trial permission
1	Documentation on the expression vector structure and exogenous inserted sequences.
2	Information on the generations of self-pollinated plants or the hybridization of each transgenic plant, along with PCR results for the target and marker genes for the respective generations or PCR results specific to the transformed plants.
3	Safety evaluation data for the recipient plant and gene donor organism, as specified in the “Guidelines for the Safety Assessment of GM Plants and Their Products for Food Use (NY/T 1101–2006).”
4	Information on the molecular and biochemical characteristics of the newly expressed protein(s), along with comparative data on amino acid sequence similarities between the new protein(s) and known toxic proteins, anti-nutritional factors, and allergens.
5	An analysis of the expressed protein’s mechanism of action against target pests in insect-resistant plants and commercially grown transgenic insect-resistant plants, along with an evaluation of cross-resistance risks.

**Table 3.** Required application documentation for an environmental release permit under China’s GMO safety governance regulations.

No.	Required application documentation for an environmental release permit
1	Documentation pertaining to intermediate trials and a summary report of the trial results.
2	A detailed description of the experimental materials used in the intermediate trials, including cultivation methods, material quantities, and agronomic characteristics.
3	Data on the integration of the exogenous sequence into the plant genome, specifying the names and generations of the test materials. These data may include Southern blot results showing target and marker gene integration and the insert’s copy number, or sequence data confirming the gene’s insertion sites and copy number, or PCR results specific to the transformed plants.
4	Transcriptional or translational level data on target gene or protein expression.
5	Information on the genetic stability of the transformed plants, including the stability of target and marker gene integration, gene expression, and the resulting phenotypic traits.
6	For transgenic plants with pest or disease resistance, methods for measuring the target protein and its content in various plant organs at different developmental stages must be provided, along with field resistance data against the target organisms.
7	Expression level data for new proteins (including target and marker gene proteins) in the edible and feed parts of the plant.
8	Research data on the cross-resistance of transgenic plants to the target pests, including comparisons with commercially cultivated resistant plants.
9	Laboratory bioassay data on potential non-target organisms. For insect-resistant transgenic plants, bioassay data for at least one non-target herbivorous species and at least two beneficial species are required. For disease-resistant transgenic plants, bioassay data for at least three non-target microorganisms are required.
10	Data on the evaluation of target traits and functional efficiency. For insect-resistant plants, the target species must be specified and indoor or field test results provided. For herbicide-resistant plants, data from tests using at least three concentration gradients of the target herbicide (recommended doses at 1×, 2×, and 4×) must be provided.
11	For herbicide-resistant transgenic plants, data on resistance to at least three other commonly used herbicides, including those typically used for the recipient plants and those to which the transgenic plants may be sensitive, must be provided.

**Table 4.** Application documents required for a production trial license under China’s GMO safety governance regulations.

No.	Necessary application data for a production trial license
1	The trial must have been conducted in a primary ecological zone appropriate for the test plants.
2	GM plant and control samples, as well as target detection methods, must be provided. The samples should include seeds of at least 99% purity, as well as details on the exogenous insertion sequence and nucleic acid detection methods that are unique to the transformed organism.
3	Data relevant to the environmental release application, along with a summary report of the environmental release results, must be submitted.
4	Detailed information on the experimental materials used in the environmental release trial must be provided, including cultivation processes, material quantities, and agronomic traits.
5	Data on the integration of the exogenous sequence into the plant genome must be submitted, including the names and generations of the test materials. These data should include Southern blot data showing the integration of exogenous fragments (e.g., transformation vector backbone, target gene, and marker gene) and the insert copy number, or sequencing results identifying the exogenous fragment’s insertion site and copy number. PCR detection profiles specific to the transformed organism should also be provided.
6	Translation level expression data for the target and marker genes’ encoded proteins, or transcriptional or translational level expression data for the target gene or protein (e.g., for genes targeted by RNAi), must be provided.
7	Genetic stability data for at least two generations of the transformed organism, including the stability of target gene integration, expression, and phenotypic traits, must be submitted.
8	Data on the survival competitiveness of the transformed organism must be provided.
9	Data on gene flow in the transgenic plant must be submitted.
10	Evaluation data on target traits and functional efficiency must be provided. For pest-resistant plants, experimental data on the seasonal occurrence of target pest damage and population dynamics in both transgenic and non-transgenic plants should be included.
11	Resistance risk assessment data for target organisms associated with disease- or pest-resistant transgenic plants must be provided.
12	Evaluation data on the transgenic plant’s effects on non-target organisms, ecosystem community structure, and pest status evolution are required.
13	In vitro stability test data on newly expressed proteins under simulated gastric digestion and heat stability conditions must be provided.
14	If necessary, comprehensive food toxicology evaluation data must be provided.
15	Testing reports from accredited technical institutions must be provided, including: 1) Nucleic acid testing to confirm the identity of the transformed organism; 2) Impact of transgenic plants (e.g., pest-resistant or drought-resistant varieties) on non-target organisms; 3) Expression levels of new proteins in edible and feed parts of the plant; and 4) Stability of newly expressed proteins under simulated gastric digestion.

**Table 5.** Application documents required for a safety certificate under China’s GMO safety governance regulations.

No.	Required application data for a safety certificate
1	A summary of data from previous experimental stages, along with a comprehensive environmental and food safety evaluation report, must be provided. At least one production test site should be established in each major ecological zone, with a total of no fewer than six environmental release and production test sites. The distance between any two test sites must be at least 300 kilometers.
2	Integration data for the exogenous sequence into the plant genome must be submitted. These data include molecular hybridization profiles that clearly indicate the exogenous fragment’s copy number (e.g., transformation vector backbone, target gene, and marker gene) or sequencing results that identify the exogenous fragment’s insertion sites and copy numbers. Full-length DNA sequences of the integrated exogenous fragment, along with both boundary sequences, must be provided, in addition to PCR detection profiles specific to the transformed organism.
3	Genetic stability data for at least three generations of the transformed organism should be provided, including the stability of target gene integration, expression, and phenotypic traits.
4	Data on the transformed organism’s survival competitiveness, natural persistence, and capacity for population establishment must be included.
5	Information on gene flow in the transgenic plant must be provided.
6	Field evaluation data on target traits and functional efficiency for at least two generations should be submitted.
7	Evaluation data on the impact of the transgenic plant on at least six non-target organisms must be provided.
8	Evaluation data on the transgenic plant’s impact on biodiversity for at least two generations, as well as a risk assessment report on its effects on ecosystem community structure and pest status evolution, should be included.
9	In addition to the results and reasoning behind the resistance risk assessment, baseline sensitivity data for target organisms must be provided for the disease- or pest-resistant substances produced by the transgenic plant. Proposed integrated pest management strategies, resistance monitoring plans, and mitigation measures must also be included.
10	Complete data on toxicity, sensitization, nutritional components, antinutritional factors, and other food safety aspects must be submitted.
11	For a re-application, commercial cultivation and environmental impact monitoring data collected during the previous approval period must be provided. For herbicide-tolerant crops, residue data for target herbicides should be included. For first-time applications, key indicators must be verified by qualified technical testing institutions.

### 2.3. Administrative Rule for the Safety of Imported Agricultural GMOs

To strengthen the safety administration of imported agricultural GMOs, the MARA issued the *Administrative Rule for the Safety of Imported Agricultural GMOs* in 2002. This rule regulates all the activities related to the importation of agricultural GMOs into China. It has been revised twice, in 2004 and 2017. The 2004 revision transferred the responsibility for overseeing the safety management of imported agricultural GMOs from the Agricultural GMO Safety Administration Office to the MARA. This shift enhanced the MARA’s authority in regulating the import safety of GMOs, thereby demonstrating the MARA’s commitment to improving safety governance. The 2017 revision introduced a key change, requiring the submission of GMO samples, control samples, and experimental materials, along with the testing methods needed for analysis. This revision primarily aimed to enhance the standardization of detection techniques for GMOs and their products.

### 2.4. Rule on the Approval of Processing of Agricultural GMOs

To strengthen the governance of imported agricultural GMOs and prevent their alteration or environmental dispersion by food and feed enterprises, the MARA issued the *Rule on the Approval of Processing of Agricultural GMOs* in 2006. The rule requires processing enterprises to maintain dedicated production lines, closed storage facilities, and waste treatment and deactivation equipment, as well as management systems to prevent the spread of GMOs. Under this rule, only food and feed enterprises approved by provincial agricultural authorities are permitted to use imported GMOs as raw materials for processing, emphasizing the agricultural sector’s primary role in GM food safety governance in China. Although the rule has been in effect for 18 years, it has not been revised. A key concern is that, while this rule governs the approval process for processing imported GMOs, China has already begun the commercial cultivation of GM food crops. Currently, no standardized procedures are in place for the use and processing of domestically grown GM crops.

### 2.5. Administrative Rule on the Labeling of Agricultural GMOs

To safeguard the consumer’s right to information and choice, while regulating the sale of agricultural GMOs and their products, the MARA issued the *Administrative Rule on the Labeling of Agricultural GMOs* in 2002. The rule mandates that any agricultural GMO and its products listed in the national GMO labeling catalog must be properly labeled before being sold within China. Products that fail to meet the labeling requirements are prohibited from being imported or sold. This rule applies to all the products included in the catalog that contain GM components, regardless of their concentration. According to the labeling regulation, GM animals and plants (including seeds, breeding livestock and poultry, and aquatic seedlings), microorganisms, products derived from GM animals, plants, or microorganisms, and products containing components of such GM organisms – such as seeds, breeding livestock and poultry, aquatic seedlings, pesticides, veterinary drugs, fertilizers, and additives – must be directly labeled as “GM XXX (name of the organism or product).” For directly processed products derived from GM agricultural products, the labeling must read either “Processed product made from GM XXX (name)” or “Produced using GM XXX (name) as raw material.” For products that are manufactured using agricultural GMOs or products containing GM ingredients, but in which GM components are no longer present or detectable in the final product, the label must read either “This product is processed from GM XXX (name), but no GM components remain in the final product,” or “This product was produced using raw materials containing GM XXX (name), but no GM components remain in the final product.” The GMO labeling must be clearly visible and printed or designed simultaneously with the product’s packaging and labeling. If it is difficult to include the GMO label directly on the existing packaging or label, an additional label may be affixed, provided that it is secure and durable. When agricultural GMOs cannot be labeled using packaging or tags, the following labeling methods may be used: (1) for agricultural GMOs sold in the food service or retail sectors in which labeling each individual product is impractical, labels may be placed on display shelves or counters, on price tags, or via signage; (2) for unpackaged and unlabeled agricultural GMOs, signage may be used at the point of sale; (3) for GMOs sold directly from transport containers without individual packaging, the container itself may be labeled, or signage may be placed nearby at the sales site; (4) if unpackaged and unlabeled GMOs cannot be labeled using signage, the seller must provide an appropriate written declaration; (5) for imported unpackaged and unlabeled GMOs for which signage is impractical, the GMO status must be indicated on customs declaration forms; (6) for GMOs subject to restricted sale or use, the scope of permitted use must be clearly stated: for example, “For sale (production, processing, or use) only in XXXX (specified scope).” The responsibility for overseeing and enforcing the labeling rule lies with local agricultural administrative departments at the county level and above. Presently, the catalog of GMOs and products subject to labeling requirements in China includes 17 products derived from 5 primary crops: soybean, corn, rapeseed, cotton seed, and tomato. These products include soybean seeds, soybeans, soybean flour, soybean oil, and soybean meal; corn seeds, corn, corn oil, and corn flour; rapeseed seeds, rapeseeds, rapeseed oil, and rapeseed meal; as well as cotton seeds, tomato seeds, fresh tomatoes, and tomato paste. Although the rule underwent minor revisions in 2004 and 2017, no substantial modifications were introduced. To improve the clarity and consistency of GMO and product labeling requirements, the MARA issued a regulatory document on agricultural GMO labeling standards in 2007. Although the administrative rule on the labeling of agricultural GMOs has undergone two rounds of revision, certain provisions remain scientifically questionable. For example, China currently enforces a strict qualitative labeling policy, which requires that any detectable presence of GMO components, regardless of concentration, must be disclosed on product labels. Given that modern detection technologies can identify GMO contents at levels as low as 0.1%, even trace amounts resulting from adventitious presence are subject to mandatory labeling. This stringent regulatory stance poses scientific challenges. During the production, transportation, storage, processing, and sale of GMO products, unintended mixing at low levels is almost unavoidable. Many countries have adopted quantitative labeling policies^18–21^ that allow minimal, technically unavoidable GMO contents in food or feed without labeling, provided that the GMO content has been safety-evaluated and approved. For example, the EU, which has expressed concerns about the safety of GM foods, has set a labeling threshold of 0.9%,^20^ whereas countries such as South Korea^22^ and Japan^23^ have set thresholds of 3% and 5%, respectively. Some countries have also exempted certain products,^24^ such as oils, starches, and food additives, which do not contain new DNA or proteins from GMOs. These exemptions are based on the refining processes effectively eliminating GMO-derived DNA and proteins. China could consider similar exemptions for GMO-free oils, starches, and related products to reduce labeling costs. Furthermore, the GMO labeling directory, established in 2002, is outdated and narrow in scope. It has not been revised to reflect the rapid growth of China’s domestic GMO industry, resulting in the exclusion of certain GM products from the labeling system. For instance, GM papaya, which is commercially grown in China, is not included in the 2002 directory. On the contrary, storage-tolerant tomato, listed in the 2002 GMO labeling catalog, has been withdrawn from the market because of their small individual fruit size and shifts in market demand. Therefore, it is essential for China to update the GMO labeling directory to include all relevant products on the market and remove those that have been withdrawn.

### 2.6. Relevant Administrative Regulatory Documents

To facilitate the commercialization of domestically developed GM food crops, the MARA revised three administrative regulatory documents in 2022: the *Rule on the Approval of Major Crop Varieties*, the *Provisions on the Naming of Agricultural Plant Varieties*, and the *Administrative Rule on Seed Production and Sales Licenses* (Table 1). The primary revision introduced specific provisions for the approval of GM food crop varieties. Under the revised rule, applications for GM food crop variety approval can only be processed by the National Crop Variety Approval Committee because provincial-level committees lack the authority to assess GM food crop varieties. Additionally, new provisions were established regarding the naming, production, and sale of GM food crop varieties. To further enhance the efficiency and standardization of the approval process, the MARA introduced national technical standards for the approval of GM corn and soybean varieties. These regulatory revisions and newly established administrative measures address critical policy gaps and thus represent a significant advancement in GM food crop commercialization in China. This regulatory update constituted a landmark reform in China’s GMO safety administrative policies.

## 3. Standardized Technical Systems for Agricultural GMO Safety Governance

The effectiveness and impacts of regulations and policies cannot be fully realized without adequate technical support. Accordingly, the MARA has developed technical standards for GMO detection and established specialized safety testing institutions. Currently, more than 40 GMO testing institutions operate across China. These institutions are accredited by national certification bodies and possess the requisite conditions and technical expertise to conduct testing on GMOs and their products. China has formulated 265 technical standards to handle the whole process of commercializing GMOs, which includes gene mining, genetic transformation, GM and gene-edited crop creation, variety breeding, safety testing, commercial cultivation, and market circulation. These standards cover three key areas: molecular characteristics, food safety, and environmental safety. The standardized testing technologies not only meet, they often exceed, international standards, thereby providing robust support for China’s GMO safety management.

## 4. China’s Agricultural GMO Safety Governance Model

**Figure 1.**
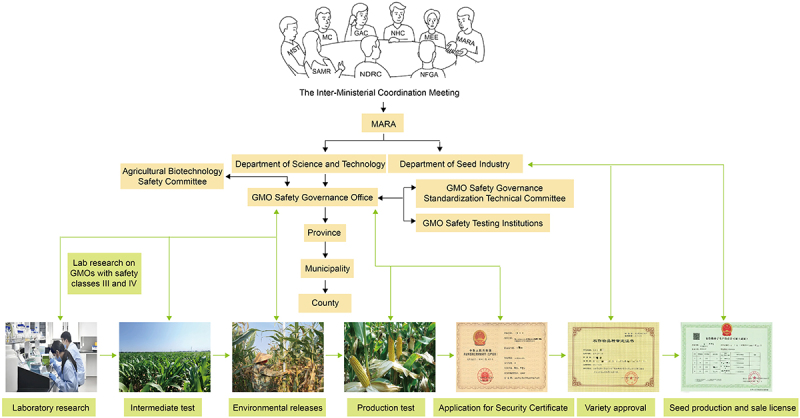
The institutional framework and governance processes for GM crop safety in China. The highest authority overseeing GMO safety is an inter-ministerial meeting under the State Council that includes several ministries, such as the Ministry of Agriculture and Rural Affairs (MARA), the Ministry of Science and Technology (MST), the Ministry of Ecology and environment (MEE), the National health Commission (NHC), the Ministry of Commerce (MC), the General Administration of Customs (GAC), the State Administration for market regulation (SAMR), the National development and Reform Commission (NDRC), and the National Forestry and grassland Administration (NFGA). The meeting’s primary responsibility is to make decisions on major GMO safety issues. The MARA is the main institution responsible for GMO safety governance, having established three technical agencies to provide support. These are the Agricultural Biotechnology safety Committee, which handles the safety evaluation of GMOs, the GMO safety governance standardization technical Committee, which develops and reviews GMO detection standards, and the GMO safety detection institution, which conducts safety testing of GMOs. GMO safety governance in China is implemented locally, with agricultural departments at the county level and above overseeing biosafety in their respective regions. GMOs are administrated through a tiered and phased approach, with the MARA directly overseeing GMOs classified as safety levels III and IV. The diagram illustrates the seven stages of GMO development, from research to commercialization: laboratory research, pilot testing, environmental release, production trials, safety certificate application, variety approval, and seed production and sales licensing. Each stage is strictly regulated, and progression is sequential, with advancement to later stages being permitted only upon completion of prior phases, unless new regulations allow otherwise. The bi-directional arrows in the research and development process diagram (at the bottom) indicate the application process and feedback. Similarly, the arrows between the GMO safety governance Office and the Agricultural Biotechnology safety Committee, as well as between the GMO safety governance Office and the GMO safety detection institution, represent task assignments and feedback on task execution results.

Over more than 30 years of exploration and practice, China has developed a GMO safety governance model tailored to its specific national context (Figure 1). This model offers a Chinese solution for balancing the commercialization of GMOs with ecological sustainability. China’s agricultural GMO safety governance is structured around a tiered system, safety evaluations, and labeling regulations. The MARA is responsible for establishing classification standards, safety evaluation criteria, and technical specifications for GMOs, while collaborating with relevant SC departments to develop GMO labeling guidelines. At the core of China’s agricultural GMO safety administration framework is the Inter-Ministerial Coordination Meeting on Agricultural GMO Safety (Figure 1), which serves as the highest decision-making body in this area. The meeting comprises heads from various SC departments, including the MARA, the Ministry of Ecology and Environment, the National Health Commission, the General Administration of Customs, the Ministry of Commerce, the MST, the State Administration for Market Regulation, the National Development and Reform Commission, and the National Forestry and Grassland Administration. This body addresses key issues related to GMO safety management. The MARA oversees the national supervision and governance of agricultural GMOs, while local governments at, or above, the county level are responsible for regulating GMOs and their products within their respective jurisdictions. The Department of Science and Technology within the MARA hosts the GMO Safety Governance Office, which manages GMO safety evaluation applications and organizes safety assessments. Additionally, the MARA has established the Agricultural Biotechnology Safety Committee, comprising experts in GMO research, production, processing, inspection, public health, and environmental protection, to evaluate GMO safety. Furthermore, the MARA has created a technical committee for the standardization of GMO safety governance. It is responsible for researching, drafting, reviewing, and promoting technical standards in this field, as well as facilitating international cooperation.

**Figure 2.**
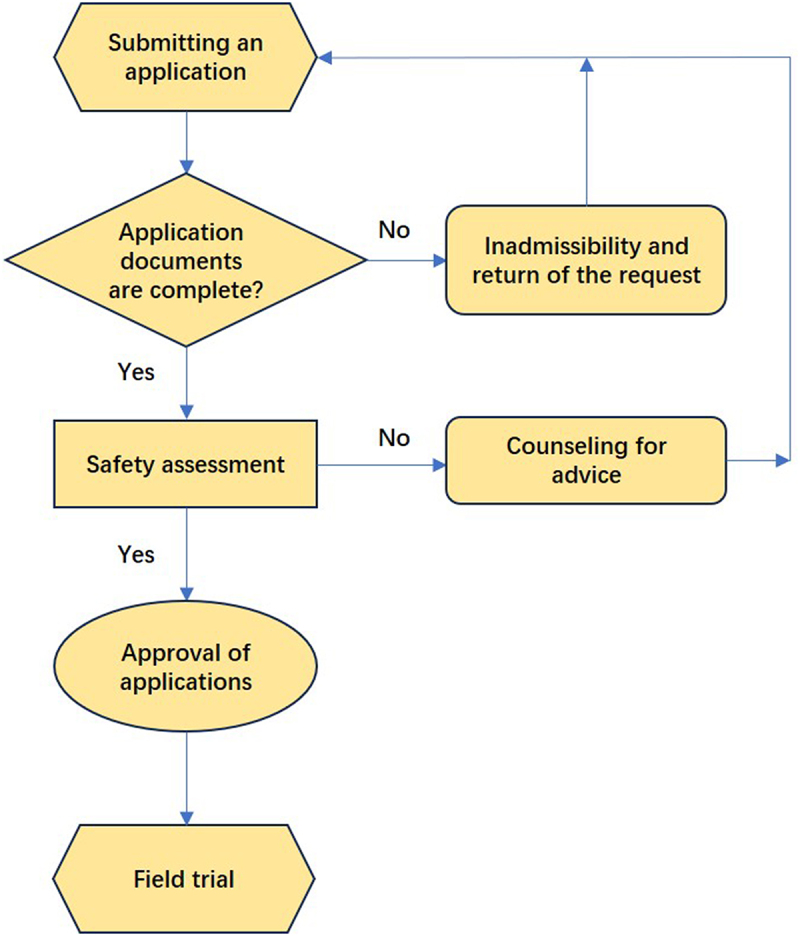
The process by which research institutions, as applicants, seek approval from the GMO safety governance Office for progression to the next testing stage, safety certification, or seed production and sales licensing. While the application process remains consistent across all the stages, the required documents and data vary. The documents and data needed for four key stages, intermediate trial, environmental release, production trials, and safety certificate application are outlined in the text and in Tables. ^4–7^

The MARA has established accredited GMO safety testing institutions to provide technical support for GMO safety governance. These institutions are required to meet the relevant qualification standards for testing and certification. During the research and development phase, laboratory research approval for Level I and II GMOs is granted by GMO safety management teams within the research institutions. For Level III and IV GMOs, approval is granted by the MARA (Figure 2). GMO trials typically progress through three stages. The first consists of intermediate trials, which are small-scale trials conducted under strict containment measures to prevent the unintended spread of GMOs. These trials are equivalent to “confined field trials” in the US regulatory framework and are primarily intended to assess the environmental safety of GMOs at a limited scale. In China, for a newly developed transgenic event, the MARA typically approves trials on a scale not exceeding 0.165 acres. These trials are followed by environmental release and production trials. After completing laboratory research, the research institution submits the required documentation to the MARA and applies for intermediate trials in experimental fields. Upon receiving approval, intermediate trials may commence. If the GMO trial needs to transition to the next stage, then the institution must submit an application along with the necessary documentation. The Agricultural Biotechnology Safety Committee (a purely technical body composed exclusively of independent academic and technical experts from fields such as scientific research, environmental protection, and biosafety assessment; the committee provides technical support to the MARA in conducting biosafety evaluations of GMOs) conducts a safety evaluation, which must be passed before the MARA approves the transition to the next stage. After the completion of production trials, the institution applies for a safety certificate by submitting the required documents to the MARA. Upon receipt of the application, the MARA delegates testing to accredited institutions and organizes a safety evaluation by the Agricultural Biotechnology Safety Committee. If the evaluation is successful, then the MARA issues the safety certificate to the research institution (Figure 2).

Once a GMO variety receives the safety certificate, variety approval may be applied for through the MARA’s Department of Seed Industry, in compliance with relevant laws and regulations. Before production can commence, a production license for any approved GMO variety must be obtained from the Department of Seed Industry. Similarly, distributors of GMO varieties must acquire a sales license issued by the same department. Throughout the research, production, processing, commercialization, import, and export of GMOs, agricultural administrative authorities at all levels implement safety governance in accordance with applicable laws and regulations. During the supervision process, if a GMO is found to pose risks to human health, animal or plant life, or the environment, or if regulatory violations have occurred, then the MARA is authorized to prohibit the research, production, processing, commercialization, or import of such GMOs. The MARA can revoke safety certificates, destroy hazardous GMOs and their products, halt unlawful activities, and impose penalties in accordance with the law. Between 2017 and 2023, 51 institutions were penalized for violating GMO safety governance regulations.^25^ These violations primarily fell into four categories: unauthorized GMO field trials and cultivation; illegal sale and mislabeling of GM seeds; unlicensed processing of GM raw materials; and the use of unapproved genetic constructs in breeding experiments. The prevalence of noncompliance with GMO safety regulations can be attributed to a combination of low legal penalties and limited regulatory capacity. For example, in cases of illegal GM crop cultivation, the maximum fine is approximately USD 6,900, an amount insufficient to serve as a meaningful deterrent. Enforcement efforts are further hampered by a shortage of personnel at the local level and infrequent sampling and testing. Economic incentives and persistent gray-market demand also play significant roles. Illegally cultivated GM maize varieties, engineered for insect and herbicide resistance, can significantly reduce production costs, encouraging farmers to engage in unauthorized plantings. In parallel, some companies circumvent official variety approval processes by illegally producing GM seeds, thereby shortening the commercialization timeline and profiting from unauthorized seed processing. Moreover, lengthy regulatory approval timelines and academic pressure constitute additional drivers of noncompliance. Field trials in China must proceed through three phases, intermediate trials, environmental release, and productive trials, but applications are accepted only twice annually, with approvals taking several months. These delays often conflict with critical agricultural timelines, prompting some research institutions to initiate trials prematurely to meet planting schedules. Limited public awareness and regulatory transparency further exacerbate these challenges. Consumer trust in GM labeling remains low, leading some businesses to intentionally omit such labels to avoid market resistance. Additionally, the lack of public access to up-to-date information on field trial activities conducted by research institutions and companies undermines public oversight and increases the risk of regulatory violations.

## 5. Research and Development of Agricultural GMOs in China

The development of agricultural biotechnology in China dates back to the 1980s (Supplementary Tables S1, S2). In 1986, China launched the *National High-Tech Research and Development Program* (1986–2016),^26^ which initiated research projects focused on developing high-quality, high-yielding, and stress-resistant plant and animal varieties through biotechnology.^27^ This marked the beginning of agricultural biotechnology research and development in the country. With the support of this program, China successfully developed and cultivated transgenic insect-resistant cotton, which now accounts for more than 75% of the total planting area, positioning China as having the world’s fifth-largest planting area of GM crops.^28^

To address fundamental scientific challenges critical to national strategic needs, China instituted the National Key Basic Research and Development Program in 1996, which was implemented continuously for 19 years (1998–2016).^29^ This program, which focused on GM technologies within the agricultural sector, resulted in several important breakthroughs. Notably, the *MOC1* gene, associated with rice tiller formation, was cloned, representing one of China’s most significant discoveries related to plant morphology, particularly to lateral branch development.^28^ This achievement has important implications for enhancing the yields of rice and other cereal crops. In addition, the successful cloning of the pig *FSH-β* gene led to the identification of a major genetic marker that influences litter size, thereby accelerating the breeding of superior pig lines.^28^ Furthermore, GM intergeneric cloned fish were successfully developed.^28^ In 1999, China launched another key national program, the *National GM Plant Research and Commercialization Program*, to accelerate research on, and the commercialization of, GM technology.^17,27^ With the support of this program, China conducted research on dozens of GM plant species. Five of these plants, insect-resistant cotton, color-altered petunia, virus-resistant tomato, storage-tolerant tomato, and virus-resistant sweet pepper, received commercialization approval. During the development of GM insect-resistant cotton, China successfully created a variety having independent intellectual property rights, making it the second country globally to possess a full set of technologies for its research and development.^27^ However, the color-altered petunia, virus-resistant tomato, storage-tolerant tomato, and virus-resistant sweet pepper have yet to be commercialized on a large scale, owing to several reasons. One key reason is persistent public concern about the potential long-term health impacts of GM technologies, which has contributed to low consumer acceptance and substantial resistance to market promotion. More importantly, limitations in China’s safety regulatory framework and associated policies have presented commercial barriers. Obtaining a safety certificate represents only the initial step toward commercial cultivation. Subsequent stages, including varietal registration and regional field trials, are also required. Initially, China’s crop variety approval procedures were designed primarily for non-GM crops and did not include specific regulatory provisions for GM plant varieties, except for GM cotton. This regulatory gap was the principal reason why some varieties with safety certificates were not commercially deployed. In addition, biotechnology companies had to weigh research and development investments against expected market returns, and in cases of uncertain profitability, commercialization was postponed. The rollout of GM plants also requires comprehensive support across the entire value chain, including seed production, processing, and labeling systems. For instance, the commercialization of virus-resistant tomatoes would require a dedicated seed supply chain. However, China’s GM plant market was initially dominated by cotton, and commercial systems for other GM crops remained underdeveloped, further complicating commercialization efforts. Furthermore, the acceptance levels of international trade partners were a consideration in the promotion of GM crops. Restrictions on GM agricultural imports in some countries could have implications for China’s export markets. Domestically, policy approaches remained cautious, with priority given to safeguarding staple food security. As a result, the commercial deployment of GM economic crops was assigned a lower policy priority. Overall, the limited commercialization of these GM varieties was the result of multiple interrelated factors, including insufficient scientific risk assessment, societal attitudes, regulatory constraints, and market-driven considerations.

In the 21st century, the development of GM food crop-breeding technologies became a central strategy for enhancing China’s agricultural competitiveness and securing a leading position in future agricultural development (Supplementary Tables S1, S2). During this period, China approved 495 intermediate trials, 237 environmental releases, and 194 production trials for GMOs.^27^ In 2008, China launched the *Major Project on Developing New GMO Varieties* (2008–2020), which garnered the highest level of financial support (USD 2.88 billion)^30^ ever allocated to an agricultural science and technology initiative. The primary objective of this project was to develop a range of new GM plant and animal varieties with disease and pest resistance, stress tolerance, high quality, high yield, and efficiency, with the ultimate goal of achieving commercialization. This project resulted in the development of 55 new transgenic cotton varieties, 3 insect-resistant poplar varieties, and 415 new lines of various crops, including rice, corn, wheat, cotton, rapeseed, and soybean.^27^ Since the initiation of the major special project on GM breeding, China has made significant advancements in areas such as identifying key functional genes, developing safe and efficient GM technologies, cultivating new crop varieties, and establishing a robust safety assurance system. These achievements have propelled China from a position of “catching up” or “keeping pace” with international developments to “leading” in several domains. In 2009, China granted safety certificates for two GM food crops: insect-resistant rice and phytase gene-modified corn.^27^ However, despite receiving safety approvals, these varieties did not proceed to commercial release. Following the completion of the *Major Project on Developing New GMO Varieties*, China launched the *Agricultural Biotechnology Major Project* in 2022. This initiative continues to prioritize GM technology as the central focus of biotechnology research and commercialization, building upon the foundation laid by the previous GM project. By 2024, China had approved 36 safety certificates for domestically developed GM food crops, including five for gene-edited crops (Table 6). A review of China’s agricultural biotechnology history revealed that GM research had progressed through five distinct phases: (1) limited innovation (1986–2000): characterized by a reliance on foreign biotechnology and the absence of insect-resistant cotton; (2) comprehensive innovation (2001–2009): defined by the launch of the *Major Project on Developing New GMO Varieties*, the largest publicly funded GMO research initiative in China’s history. Nearly all the key agricultural ministries and national-level research institutions were engaged in a coordinated national effort to advance genetic engineering technologies. During this period, China granted five safety certificates for GM events, including those for cotton, corn, and rice (Table 6). However, owing to extended research timelines, public skepticism, and limitations in biosafety regulatory policies, many research outcomes did not materialize until after this phase (Table 6); (3) early-warning management (2010–2013): during this period, the development and potential commercialization of GMOs in China faced significant challenges because of growing domestic and international opposition, fueled by widespread public skepticism and negative media coverage. Government agencies did not respond promptly with effective public communication or science-based outreach to address misinformation about GMOs. As a result, public sentiment, shaped by a lack of scientific understanding, turned increasingly resistant. To ease public concern and maintain social stability, the agricultural authorities temporarily suspended the approval and issuance of new GMO safety certificates. In this context, China’s GMO governance entered what can be described as an “early-warning management” phase, characterized by heightened caution and a regulatory pause in response to perceived social risks; (4) cautious promotion (2014–2020): defined by the Chinese governments organization of extensive outreach efforts to improve the public’s understanding of GMOs. Experts, scholars, and researchers from universities, scientific institutions, and biosafety regulatory agencies were mobilized to conduct frequent online and offline science communication activities aimed at guiding the public toward a more rational and evidence-based perception of GMOs. As a result, public attitudes toward GMOs gradually shifted in a more scientific and measured direction. To avoid triggering renewed public concern during this delicate transition, the government adopted a cautious approach to GMO deployment. Six safety certificates were approved during this period, including for GM corn and soybean. However, commercial cultivation was not initiated, largely because of the absence of a clear regulatory framework for the varietal approval of GM corn and soybean; and (5) accelerating the commercialization of domestically developed GM food crops (2021–present): following the cautious and exploratory approach of the previous phase, a more favorable public discourse toward GMOs began to emerge in China. Coupled with the rising domestic demand for feed corn and soybean driven by the expanding livestock and poultry industries, the government moved to accelerate the commercialization of domestically developed GM food crops. During this period, China initiated pilot programs and demonstration projects for the commercial cultivation of GM corn and soybean varieties. Throughout these stages, China has developed a series of high-yield, high-quality, pest-resistant, stress-tolerant, and efficient new varieties.^31^ These achievements aim to reduce reliance on imported GM corn and soybean, while accelerating the commercialization of domestically developed GM food crops through the development and accumulation of improved varieties and advanced technologies.

**Table 6.** Transformant’s developed and safety-certified in China (1997–2024).

No.	Transformant	Crop	Applicant Institution	Exogenous Gene(s)	Trait Improvement	Date of Safety Certificate
1	Bt cotton GK-12	Cotton	Institute of Biotechnology, Chinese Academy of Agricultural Sciences	*Cry1A*	Insect resistance	July 1, 1997
2	Bt cotton Jinmian-26	Cotton	Institute of Biotechnology, Chinese Academy of Agricultural Sciences	*GFM Cry1A*	Insect resistance	July 1, 1997
3	Bt cotton SGK-321	Cotton	Institute of Biotechnology, Chinese Academy of Agricultural Sciences	*CpTI, Cry1A*	Insect resistance	July 1, 1997
4	Bt cotton DR409	Cotton	Institute of Microbiology, Chinese Academy of Sciences and Cotton Institute, Shaanxi Academy of Agricultural Sciences	*Bt, API*	Insect resistance	December 20, 2003
5	Herbicide-tolerant cotton GGK2	Cotton	Xinjiang Guoxin Seed Industry Co., Ltd and Institute of Biotechnology, Chinese Academy of Agricultural Sciences	*gr79epsps, gat*	Herbicide tolerance	January 2, 2024
6	Herbicide-tolerant cotton KJC017	Cotton	Kejidalong (Beijing) Biotechnology Co., Ltd.	*cp4epsps*	Herbicide tolerance	October 8, 2024
7	Virus-resistant Papaya YK1601	Papaya	Institute of Tropical Agriculture, Chinese Academy of Sciences	PRSV-YK CP	Resistance to Papaya ringspot virus	December 20, 2018
8	Virus-resistant papaya Huannong 1	Papaya	South China Agricultural University	Virus replication enzyme gene of ringspot viruses	Resistance to Papaya ringspot virus	July 20, 2006
9	Phytase-enhanced corn BVLA430101	Corn	Institute of Biotechnology, Chinese Academy of Agricultural Sciences	Phytase gene	Enhanced phytase production	August 17, 2009
10	Insect-resistant, herbicide-tolerant corn DBN9936	Corn	Dabeinong Biotechnology Co., Ltd.	*Cry1Ab, epsps*	Resistance to Lepidoptera pests and glyphosate	December 2, 2019
11	Insect-resistant, herbicide-tolerant corn Ruifeng 125	Corn	Hangzhou Ruifeng Biotechnology Co., Ltd.	*Cry1Ab/Cry2Aj, g10evo-epsps*	Resistance to Lepidoptera pests and glyphosate	December 2, 2019
12	Herbicide-tolerant corn DBN9858	Corn	Dabeinong Biotechnology Co., Ltd.	*epsps, pat*	Glyphosate and glufosinate resistance	June 11, 2020
13	Insect-resistant, herbicide-tolerant corn DBN9501	Corn	Dabeinong Biotechnology Co., Ltd.	*Vip3Aa19, pat*	Resistance to Lepidoptera pests and glufosinate	December 29, 2020
14	Insect-resistant corn ND207	Corn	China National Forestry Seed Group Corporation; China Agricultural University	*mcry1Ab, mcry2Ab*	Insect resistance	December 17, 2021
15	Insect-resistant corn Zheda Ruifeng 8	Corn	Hangzhou Ruifeng Biotechnology Co., Ltd.	*Cry1Ab, Cry2Ab*	Insect resistance	December 17, 2021
16	Insect-resistant, herbicide-tolerant corn DBN3601T	Corn	Dabeinong Biotechnology Co., Ltd.	*Cry1Ab, epsps, vip3Aa19, pat*	Insect resistance, herbicide tolerance	December 17, 2021
17	Herbicide-tolerant corn nCX-1	Corn	Hangzhou Ruifeng Biotechnology Co., Ltd.	*CdP450, cp4epsps*	Herbicide tolerance	April 22, 2022
18	Insect-resistant, herbicide-tolerant corn Bt11×GA21	Corn	China National Seed Group Corporation	*Cry1Ab, pat, mepsps*	Insect resistance, herbicide tolerance	April 22, 2022
19	Insect-resistant and herbicide-tolerant corn Bt11×MIR162×GA21	Corn	China National Seed Group Corporation	*Cry1Ab, pat, Vip3Aa20, mepsps*	Insect resistance, herbicide tolerance	April 22, 2022
20	Herbicide-tolerant corn GA21	Corn	China National Seed Group Corporation	*mepsps*	Herbicide tolerance	April 22, 2022
21	Insect-resistant and herbicide-tolerant corn BFL4–2	Corn	Yuan Longping High-tech Agriculture Co., Ltd. and Institute of Biotechnology, Chinese Academy of Agricultural Sciences	*Cry1Ab, Cry1F, cp4epsps*	Insect resistance, herbicide tolerance	January 5, 2023
22	Herbicide-tolerant corn CC-2	Corn	China National Forestry Seed Group Corporation; China Agricultural University	*maroACC*	Herbicide tolerance	January 5, 2023
23	Insect-resistant, herbicide-tolerant corn Zheda Ruifeng 8×nCX-1	Corn	Hangzhou Ruifeng Biotechnology Co., Ltd.	*Cry1Ab, Cry2Ab, CdP450, cp4epsps*	Insect resistance, herbicide tolerance	January 2, 2024
24	Insect-resistant, herbicide-tolerant corn Ruifeng 125×nCX-1	Corn	Hangzhou Ruifeng Biotechnology Co., Ltd.	*Cry1Ab/Cry2Aj, g10evo-epsps, CdP450, cp4epsps*	Insect resistance, herbicide tolerance	January 2, 2024
25	Insect-resistant, herbicide-tolerant corn LP026–2	Corn	Longping Biotechnology (Hainan) Co., Ltd.	*Cry2Ab, Cry1Fa, Cry1Ab, epsps*	Insect resistance, herbicide tolerance	January 2, 2024
26	Herbicide-tolerant Corn LW2–1	Corn	Longping Biotechnology (Hainan) Co., Ltd.	*epsps, pat*	Herbicide tolerance	January 2, 2024
27	Herbicide-tolerant corn WYN17132	Corn	Zhejiang Xin’an Chemical Group Co., Ltd.	*am79epsps*	Herbicide tolerance	January 2, 2024
28	Insect-resistant, herbicide-tolerant corn WYN041	Corn	Zhejiang Xin’an Chemical Group Co., Ltd.	*Cry1Ab, am79epsps*	Insect resistance, herbicide tolerance	January 2, 2024
29	Insect-resistant, herbicide-tolerant corn BBL2–2	Corn	Beijing Aoruijin Seed Industry Co., Ltd.; Institute of Biotechnology, Chinese Academy of Agricultural Sciences; Beijing Boaiyuan Biotechnology Co., Ltd.	*Cry1Ab, Cry3Bb, cp4epsps*	Insect resistance, herbicide tolerance	May 7, 2024
30	Herbicide-tolerant soybean SHZD3201	Soybean	Shanghai Jiao Tong University	*g10evo-epsps*	Glyphosate resistance	December 2, 2019
31	Herbicide-tolerant soybean Zhonghuang 6106	Soybean	Institute of Crop Science, Chinese Academy of Agricultural Sciences	*g2-epsps, gat*	Glyphosate resistance	June 11, 2020
32	Herbicide-tolerant soybean DBN9004	Soybean	Beijing Dabeinong Biotechnology Co., Ltd.	*epsps, pat*	Glyphosate and glufosinate resistance	December 29, 2020
33	Insect-resistant soybean CAL16	Soybean	Hangzhou Ruifeng Biotechnology Co., Ltd.	*Cry1Ab/Vip3Da*	Insect resistance	January 5, 2023
34	Insect-resistant, herbicide-tolerant soybean DBN8002	Soybean	Beijing Dabeinong Biotechnology Co., Ltd.	*mvip3Aa, pat*	Insect resistance, herbicide tolerance	April 21, 2023
35	Herbicide-tolerant soybean WYN341GmC	Soybean	Zhejiang Xin’an Chemical Group Co., Ltd.	*cp4epsps*	Herbicide tolerance	January 2, 2024
36	Herbicide-tolerant soybean WYN029GmA	Soybean	Zhejiang Xin’an Chemical Group Co., Ltd.	*mam79epsps*	Herbicide tolerance	January 2, 2024
37	Quality trait-improved soybean AE15–18–1	Soybean	Shandong Shunfeng Biotechnology Co., Ltd.	Mutated *gmfad2-1a, gmfad2-1b*	Quality trait improvement	April 21, 2023
38	Physiological trait-improved soybean 25T93–1	Soybean	Shandong Shunfeng Biotechnology Co., Ltd.	Mutated *GmELF3a*	Physiological trait improvement	January 2, 2024
39	Quality trait-improved soybean P16	Soybean	Suzhou Qi He Biotechnology Co., Ltd.	Mutated *GmFAD2-1A, GmFAD2-1B*	Quality trait improvement	January 2, 2024
40	Yield trait-improved corn 179AC19–13–13	Corn	Shandong Shunfeng Biotechnology Co., Ltd.	Mutated *Br2*	Yield trait improvement	May 7, 2024
41	Disease-resistant wheat MLO-KNRNP	Wheat	Suzhou Qi He Biotechnology Co., Ltd.; Institute of Genetics and Developmental Biology, Chinese Academy of Sciences	Mutated *TaMLO-A1, TaMLO-B1, TaMLO-D1, TaMLOX*	Disease resistance	May 7, 2024
42	Insect-resistant rice Huahui 1 (TT51–1)	Rice	Huazhong Agricultural University	*Cry1Ab/Ac*	Resistance to Lepidoptera pests	August 17, 2009
43	Insect-resistant hybrid rice Bt Shanyou 63	Rice	Huazhong Agricultural University	*Cry1Ab/Ac*	Resistance to Lepidoptera pests	August 17, 2009

### 5.1. GM Food Crop Safety Certificates Approved by China

From 1997 to 2024, China granted 43 safety certificates for domestically developed GM crops.^32^ The recipients included insect-resistant cotton, insect-resistant rice, herbicide-tolerant corn, GM high-phytase corn, yield-enhanced corn, insect-resistant soybean, herbicide-tolerant soybean, quality-enhanced soybean, virus-resistant papaya, and disease-resistant wheat (Table 6). This total does not include derivative lines of GMs or gene-edited crops. Specifically, insect-resistant rice received two safety certificates, whereas insect-resistant, herbicide-tolerant, and high-phytase corn, as well as yield-enhanced corn, collectively obtained 21 safety certificates (including one for gene-edited corn). Herbicide-tolerant, quality-enhanced soybean received 10 safety certificates (three of which were for gene-edited soybean), and gene-edited disease-resistant wheat received one safety certificate. In 1997, China issued its first safety certificate for an insect-resistant cotton variety, but the same crop received only one additional certificate over the next 6 years. Subsequently, the government approved one safety certificate for a virus-resistant papaya in 2006 and three certificates in 2009 for two insect-resistant rice and one phytase-enhanced corn (Table 6). Over the following 8 years (2010–2017), however, no additional safety certificates for GM crops were approved by the agricultural authorities. As noted earlier, this period coincided with heightened domestic and international public debate over GMO safety, which significantly influenced China’s policy environment for GMO research and commercialization. During this time, China’s GMO safety governance was characterized by a combination of “early-warning management” and “cautious promotion,” resulting in an overall tightening of regulatory policy. Notably, no GMO safety certificates were granted for an entire 8-year period. By contrast, the majority of GMO safety certificates have been issued during the current phase of accelerating commercialization. Since 2021, 25 certificates have been granted, including for GM corn and soybean, accounting for 58% of all certificates issued to date (Table 6). This surge may be attributed to the following two key factors: the cumulative impact of long-term government investment in GMO research and a gradual shift toward a more rational public perception of GMO safety. It is worth noting that although GM rice received safety certification in 2009, China has yet to approve any GM rice varieties for commercial cultivation. This reflects China’s prioritized commercialization strategy for GM crops, which began with non-food GM economic crops (such as cotton), followed by GM crops for indirect human consumption (such as feed corn and soybean), and eventually progressed to GM crops for direct human consumption, such as rice and wheat. This strategy is more conservative than those of countries such as the United States and Brazil, primarily because of safety concerns surrounding rice, a staple food in China. This decision embodies the government’s multifaceted concerns regarding the commercialization of GM staple crops, particularly GM rice. Compared with public opinion prior to 2010, societal attitudes in China have become more favorable toward the commercialization of GMOs. However, rice remains the primary staple food for the Chinese population and is directly linked to the daily dietary safety of 1.4 billion people. Notably, no country in the world has yet approved GM rice for large-scale commercial cultivation as a staple crop, whereas GM maize and soybean have a commercial history spanning more than 30 years. Concerns about the potential long-term health effects of consuming GM rice persist among the public. As a staple food, the adoption of GM rice also risks triggering cultural resistance, especially given the symbolic role of rice in Chinese agriculture. Conversely, GM maize and soybean face relatively fewer barriers to adoption because they are primarily used for feed and industrial processing. In addition, the rapid advancement of gene-editing technologies offers a new pathway for improving China’s staple crops. Unlike traditional GM approaches, gene editing does not involve the introduction of foreign genes, allows for shorter approval timelines, and tends to be more acceptable to the public. For instance, the approval of a gene-edited wheat variety with resistance to powdery mildew in 2024 may signal a policy shift in China toward the broader application of biotechnology in staple crop development.

### 5.2. GM Food Crop Varieties Approved by China

In 2023, China simultaneously approved both safety certificates and variety registrations for GM food crops. To date, 81 GM food crop varieties (Table 7) have been approved, including 64 for corn and 17 for soybean^33^ and.^34^ Additionally, 85 seed companies have been granted licenses for the production and commercialization of GM food crop seeds,^35^ signifying the final regulatory step toward the industrialization of GM food crops in China. The 81 approved varieties were primarily developed by backcrossing insect-resistant, herbicide-tolerant, or combined insect-resistant and herbicide-tolerant, transgenic lines with locally dominant non-GM varieties. Their key traits include insect resistance, herbicide tolerance, or a combination. Most transgenic lines contain two to four genes that confer these traits (Table 7). Of the 21 corn transgenic lines that received safety certificates, 7 were backcrossed into 64 local commercial corn varieties. These seven lines include three developed by Beijing Dabeinong Biotechnology Co., Ltd., containing the *cry1Ab* and *epsps* genes for insect resistance and herbicide tolerance (DBN9936), respectively, the *epsps* and *pat* genes for herbicide tolerance (DBN9858), and the *cry1Ab, epsps, vip3Aa19*, and *pat* genes for both traits (DBN3601T). One line (Ruifeng125), developed by Hangzhou Ruifeng Biotechnology Co., Ltd. and Zhejiang University, contains the *cry1Ab/cry2Aj* and *g10evo-epsps* genes for insect resistance and herbicide tolerance, respectively. Another line (ND207), developed by Beijing Liangyuan Biotechnology Co., Ltd., contains the *mcry1Ab* and *mcry2Ab* genes for insect resistance. One line, developed by China National Seed Group Co., Ltd., contains the *cry1Ab* gene for insect resistance and the *pat* and *mepsps* genes for herbicide tolerance (Bt11×GA21) and another contains *cry1Ab*, *pat*, *vip3Aa20*, and *mepsps* genes for the same traits (Bt11×MIR162×GA21). Among the 7 soybean transgenic lines that received safety certificates, 2 were backcrossed into 17 local soybean varieties. One line was developed by Beijing Dabeinong Biotechnology Co., Ltd. and contains the *epsps* and *pat* genes for glyphosate and glufosinate tolerance, respectively (DBN9004), whereas the other was developed by the Institute of Crop Science, Chinese Academy of Agricultural Sciences and contains the *g2-epsps* and *gat* genes for glyphosate tolerance (Zhonghuang6106). Beijing Dabeinong Biotechnology Co., Ltd. developed 88.9% of the approved transgenic lines. Among them, DBN9936 was present in 95.3% of the approved corn variety lineages, whereas DBN9004 was present in for 35.3% of the soybean variety lineages, underscoring Beijing Dabeinong’s leading role in China’s GM biotechnology-related research and development.

**Table 7.** GM corn and soybean varieties developed and approved in China (2023–2024).

No.	Variety Name	Crop	Breeding Institution	Parental Lines	Transgenic Traits	Transformant	Transformant Owner
1	Yufeng 303D	Corn	Beijing Lianchuang Seed Industry Co., Ltd.	CT1699 × CT3354 (DBN9936)	Resistance to Asian corn borer; glyphosate herbicide tolerance	DBN9936	Beijing Dabeinong Biotechnology Co., Ltd.
2	Zhongkeyu 505D	Corn	Beijing Lianchuang Seed Industry Co., Ltd.	CT1668 × CT3354 (DBN9936)	Resistance to Asian corn borer and armyworm; glyphosate herbicide tolerance	DBN9936	Beijing Dabeinong Biotechnology Co., Ltd.
3	Jiaxi 100D	Corn	Beijing Lianchuang Seed Industry Co., Ltd.	CT61253 × CT3351 (DBN9936)	Resistance to Asian corn borer; glyphosate herbicide tolerance	DBN9936	Beijing Dabeinong Biotechnology Co., Ltd.
4	Zhongkeyu 505R	Corn	Beijing Lianchuang Seed Industry Co., Ltd.	CT1668 × CT3354 (Ruifeng 125)	Resistance to Asian corn borer	Ruifeng 125	Hangzhou Ruifeng Biotechnology Co., Ltd., Zhejiang University
5	Yufeng 303R	Corn	Beijing Lianchuang Seed Industry Co., Ltd.	CT1669 × CT3354 (Ruifeng 125)	Resistance to Asian corn borer	Ruifeng 125	Hangzhou Ruifeng Biotechnology Co., Ltd., Zhejiang University
6	Yufeng 303H	Corn	Beijing Lianchuang Seed Industry Co., Ltd.	CT1669 × CT3354 (DBN9858)	Resistance to Asian corn borer; glufosinate herbicide tolerance	DBN9858	Beijing Dabeinong Biotechnology Co., Ltd.
7	Jingke 968TK	Corn	Corn Research Institute, Beijing Academy of Agriculture and Forestry Sciences	Jing724 × Jing92 (Ruifeng 125)	Resistance to Asian corn borer	Ruifeng 125	Hangzhou Ruifeng Biotechnology Co., Ltd., Zhejiang University
8	Jingke 968D	Corn	Corn Research Institute, Beijing Academy of Agriculture and Forestry Sciences	Jing724 (DBN9936) × Jing92	Resistance to Asian corn borer and armyworm; glyphosate herbicide tolerance	DBN9936	Beijing Dabeinong Biotechnology Co., Ltd.
9	Zhengdan 958D	Corn	Beijing Fengdu High-tech Seed Industry Co., Ltd.	Zheng58 (DBN9936) × Chang7–2	Resistance to Asian corn borer; glyphosate herbicide tolerance	DBN9936	Beijing Dabeinong Biotechnology Co., Ltd.
10	Nonghua 803D	Corn	Beijing Fengdu High-tech Seed Industry Co., Ltd.	K4104–16 × B8328 (DBN9936)	Resistance to armyworm	DBN9936	Beijing Dabeinong Biotechnology Co., Ltd.
11	Nongda 372R	Corn	Hebei Xuntian Agricultural Technology Co., Ltd.	X24621 (Ruifeng 125) × BA702	Resistance to Asian corn borer, armyworm, and cotton bollworm	Ruifeng 125	Hangzhou Ruifeng Biotechnology Co., Ltd.
12	Zhengdan 958K	Corn	Shanxi Zhongnong Saibo Seed Industry Co., Ltd.	Zheng58 (ND207) × Chang7–2	Resistance to Asian corn borer	ND207	Beijing Liangyuan Biotechnology Co., Ltd.
13	Ruipu 909D	Corn	Shanxi Agricultural University and Shanxi Sanlian Modern Seed Industry Technology Co., Ltd.	RP86 (DBN9936) × RP06	Resistance to Asian corn borer and armyworm; glyphosate herbicide tolerance	DBN9936	Beijing Dabeinong Biotechnology Co., Ltd.
14	Dafeng 30F	Corn	Shanxi Dafeng Seed Industry Co., Ltd.	A311 (DBN9936) × PH4CV	Resistance to Asian corn borer and armyworm; glyphosate herbicide tolerance	DBN9936	Beijing Dabeinong Biotechnology Co., Ltd.
15	Lihe 1D	Corn	Inner Mongolia Lihe Agricultural Technology Development Co., Ltd.	M1001 (DBN9936) × F2001	Resistance to Asian corn borer and armyworm; glyphosate herbicide tolerance	DBN9936	Beijing Dabeinong Biotechnology Co., Ltd.
16	Kehe 699D	Corn	Inner Mongolia Bayannaoer Kehe Seed Industry Co., Ltd.	KH636 × KH766 (DBN9936)	Resistance to Asian corn borer and armyworm; glyphosate herbicide tolerance	DBN9936	Beijing Dabeinong Biotechnology Co., Ltd.
17	Dongdan 1331D	Corn	Liaoning Dongya Seed Industry Co., Ltd.	XC2327 × XB1621 (DBN9936)	Resistance to Asian corn borer and armyworm; glyphosate herbicide tolerance	DBN9936	Beijing Dabeinong Biotechnology Co., Ltd.
18	Dongdan 1331K	Corn	Liaoning Dongya Seed Industry Co., Ltd.	XC2327 × XB1621 (ND207)	Resistance to Asian corn borer	ND207	Beijing Liangyuan Biotechnology Co., Ltd.
19	Hongshuo 899SK	Corn	Liaoning Hongshuo Seed Industry Technology Co., Ltd.	D5433 (DBN9936) × T36	Resistance to Asian corn borer; glyphosate herbicide tolerance	DBN9936	Beijing Dabeinong Biotechnology Co., Ltd.
20	Xiangyu 998HZ	Corn	Jilin Hongxiang Agricultural Group Hongxiang Seed Industry Co., Ltd.	Y822 (Ruifeng 125) × X9231	Resistance to Asian corn borer	Ruifeng 125	Hangzhou Ruifeng Biotechnology Co., Ltd., Zhejiang University
21	Youdi 998HZ	Corn	Jilin Hongxiang Agricultural Group Hongxiang Seed Industry Co., Ltd.	JL712 (Ruifeng 125) × JL715	Resistance to Asian corn borer	Ruifeng 125	Hangzhou Ruifeng Biotechnology Co., Ltd., Zhejiang University
22	Tianyu 108Z	Corn	Jilin Yuntianhua Seed Industry Technology Co., Ltd.	YTH001 (ND207) × TCB01	Resistance to Asian corn borer	ND207	Beijing Liangyuan Biotechnology Co., Ltd.
23	Zengyu 1572KK	Corn	Tieling Zengyu Seed Technology Research Co., Ltd.	11A341 × Y1217 (DBN9936)	Resistance to Asian corn borer; glyphosate herbicide tolerance	DBN9936	Beijing Dabeinong Biotechnology Co., Ltd.
24	Denghai 605D	Corn	Shandong Denghai Seed Industry Co., Ltd.	DH351 × DH382 (DBN9936)	Resistance to Asian corn borer; glyphosate herbicide tolerance	DBN9936	Beijing Dabeinong Biotechnology Co., Ltd.
25	Denghai 533D	Corn	Shandong Denghai Seed Industry Co., Ltd.	Denghai 22 × DH382 (DBN9936)	Resistance to Asian corn borer; glyphosate herbicide tolerance	DBN9936	Beijing Dabeinong Biotechnology Co., Ltd.
26	Zhengdan 958GK	Corn	Henan Fujitai Seed Industry Co., Ltd.	Zheng58 (DBN9936) × Chang7–2	Resistance to Asian corn borer and armyworm	Ruifeng 125	Hangzhou Ruifeng Biotechnology Co., Ltd., Zhejiang University
27	Jinyuan 177K	Corn	Henan Jinyuan Seed Industry Co., Ltd.	JCY16667 × JCY16557 (ND207)	Resistance to Asian corn borer	ND207	Beijing Liangyuan Biotechnology Co., Ltd.
28	Jingke 986GE	Corn	Henan Modern Seed Industry Co., Ltd.	Jing724A × Jing92 (Ruifeng 125)	Resistance to Asian corn borer and armyworm	Ruifeng 125	Hangzhou Ruifeng Biotechnology Co., Ltd., Zhejiang University
29	Kangnong 20065KK	Corn	Hubei Kangnong Seed Industry Co., Ltd.	FL335 (DBN9936) × FL11646	Resistance to Asian corn borer, armyworm and cotton bollworm; glyphosate herbicide tolerance	DBN9936	Beijing Dabeinong Biotechnology Co., Ltd.
30	Huimin 207R	Corn	Hubei Huimin Agricultural Technology Co., Ltd.	H1 (Ruifeng 125) × M1	Resistance to Asian corn borer and armyworm	Ruifeng 125	Hangzhou Ruifeng Biotechnology Co., Ltd., Zhejiang University
31	Yuanke 105WG	Corn	China National Seed Group Corporation	H7–5 (Bt11 × GA21) × Y2A	Resistance to Asian corn borer and armyworm; glyphosate herbicide tolerance	Bt11 × GA21	China National Seed Group Corporation
32	Yuanke 105D	Corn	China National Seed Group Corporation	H7–5 (DBN9936) × Y2A	Resistance to Asian corn borer and armyworm; glyphosate herbicide tolerance	DBN9936	Beijing Dabeinong Biotechnology Co., Ltd.
33	Heyu 187D	Corn	China National Seed Group Corporation	V76–1 (DBN9936) × WC009	Resistance to Asian corn borer and armyworm; glyphosate herbicide tolerance	DBN9936	Beijing Dabeinong Biotechnology Co., Ltd.
34	Xianda 901ZL	Corn	China National Seed Group Corporation	NP5024 (Bt11 × MIR162 × GA21) × NP5063	Resistance to Asian corn borer, armyworm, cotton bollworm and *Spodoptera frugiperda*; glyphosate and glufosinate herbicide tolerance	Bt11 × MIR162 × GA21	China National Seed Group Corporation
35	Tie 391K	Corn	Sichuan Tonglu Agricultural Technology Co., Ltd.	T1004 (DBN9936) × T12067	Resistance to Asian corn borer, armyworm; glyphosate herbicide tolerance	DBN9936	Beijing Dabeinong Biotechnology Co., Ltd.
36	Luodan 566DT	Corn	Yunnan Datian Seed Industry Co., Ltd.	703 (DBN3601T) × 3731	Resistance to Asian corn borer; glyphosate herbicide tolerance	DBN3601T	Beijing Dabeinong Biotechnology Co., Ltd.
37	Wugu 3861KK	Corn	Gansu Wugu Seed Industry Co., Ltd.	WG6320 (DBN3601T) × WG646	Resistance to Asian corn borer, armyworm and cotton bollworm; glyphosate and glufosinate herbicide tolerance	DBN3601T	Beijing Dabeinong Biotechnology Co., Ltd.
38	Hengfeng 728D	Corn	Beijing Huannong Weiye Seed Technology Co., Ltd.	B2817 (DBN9936) × B238	Resistance to Asian corn borer; glyphosate herbicide tolerance	DBN9936	Beijing Dabeinong Biotechnology Co., Ltd.
39	Leying 797D	Corn	Beijing Huannong Weiye Seed Technology Co., Ltd.	B3191 × B609 (DBN9936)	Resistance to Asian corn borer and armyworm; glyphosate herbicide tolerance	DBN9936	Beijing Dabeinong Biotechnology Co., Ltd.
40	Shengmei 999D	Corn	Beijing Huannong Weiye Seed Technology Co., Ltd.	B2817 × B609 (DBN9936)	Resistance to Asian corn borer and armyworm; glyphosate herbicide tolerance	DBN9936	Beijing Dabeinong Biotechnology Co., Ltd.
41	Yufeng 623K	Corn	Chengde Yufeng Seed Industry Co., Ltd.	CX207 × C×242(ND207)	Resistance to Asian corn borer and armyworm	ND207	Beijing Liangyuan Biotechnology Co., Ltd.
42	Tiannong 9D	Corn	Fushun Tiannong Seed Industry Co., Ltd.	T106 (DBN9936) × W08	Resistance to Asian corn borer and armyworm; glyphosate herbicide tolerance	DBN9936	Beijing Dabeinong Biotechnology Co., Ltd.
43	Tianyu 616K	Corn	Jilin Yuntianhua Seed Industry Co., Ltd.	YTH002 × TCB01 (ND207)	Resistance to Asian corn borer and armyworm	DBN9936	Beijing Dabeinong Biotechnology Co., Ltd.
44	Liaoke 38K	Corn	Beidahuang Kenfeng Seed Industry Co., Ltd.	H32 × h45 (ND207)	Resistance to Asian corn borer and armyworm	DBN9936	Beijing Dabeinong Biotechnology Co., Ltd.
45	Liaoke 38D	Corn	Beidahuang Kenfeng Seed Industry Co., Ltd.	H32 (DBN9936) × h45	Resistance to Asian corn borer and glyphosate herbicide tolerance	DBN9936	Beijing Dabeinong Biotechnology Co., Ltd.
46	Jingke 968K	Corn	Corn Research Institute, Beijing Academy of Agriculture and Forestry Sciences	Jing724 (ND207) × Jing92	Resistance to Asian corn borer and armyworm	ND207	Beijing Liangyuan Biotechnology Co., Ltd.
47	Jinke Yu 3306D	Corn	Shanxi Dafeng Seed Industry Co., Ltd.	N16082 (DBN9936) × X1267	Resistance to Asian corn borer; glyphosate tolerance	DBN9936	Beijing Dabeinong Biotechnology Co., Ltd.
48	Huxin 358D	Corn	Huludao New Variety Sci-Tech Development Co., Ltd.	H9–1 (DBN9936) × h3–4	Resistance to Asian corn borer and armyworm; glyphosate tolerance	DBN9936	Beijing Dabeinong Biotechnology Co., Ltd.
49	Rongyu 8K	Corn	Huludao New Variety Sci-Tech Development Co., Ltd.	C13 (ND207) × 9818	Resistance to Asian corn borer and armyworm	ND207	Beijing Liangyuan Biotechnology Co., Ltd.
50	Hongkai 706D	Corn	Huludao New Variety Sci-Tech Development Co., Ltd.	H9–1 (DBN9936) × hL7–8	Resistance to Asian corn borer and armyworm; glyphosate tolerance	DBN9936	Beijing Dabeinong Biotechnology Co., Ltd.
51	Hengyu 1D	Corn	Jilin Yuanke Agricultural Development Co., Ltd.	Z47 (DBN9936) × Dian49	Resistance to Asian corn borer and armyworm; glyphosate tolerance	DBN9936	Beijing Dabeinong Biotechnology Co., Ltd.
52	Longken 1755K	Corn	Beidahuang Kenfeng Seed Industry Co., Ltd.	X5802 × DK301 (ND207)	Resistance to Asian corn borer and armyworm	ND207	Beijing Liangyuan Biotechnology Co., Ltd.
53	Aomei 95D	Corn	Sichuan Tonglu Agricultural Sci-Tech Co., Ltd.	T8 (DBN9936) × T169	Resistance to Asian corn borer and armyworm; glyphosate tolerance	DBN9936	Beijing Dabeinong Biotechnology Co., Ltd.
54	Heyu 185WG	Corn	China National Seed Group Corporation	TH751 (Bt11 × GA21) × TH19A	Resistance to Asian corn borer and armyworm	Bt11 × GA21	China National Seed Group Corporation
55	Lianchuang 808R	Corn	Beijing Lianchuang Seed Co., Ltd.	CT3566 × CT3354 (Ruifeng 125)	Resistance to Asian corn borer	Ruifeng 125	Hangzhou Ruifeng Biotechnology Co., Ltd.
56	Jingnongke 728K	Corn	Corn Research Institute, Beijing Academy of Agricultural and Forestry Sciences	JingMC01 × Jing2416 (ND207)	Resistance to Asian corn borer, armyworm and cotton bollworm	ND207	Beijing Liangyuan Biotechnology Co., Ltd.
57	Dedan 123R	Corn	Denong Seed Co., Ltd.	CA24 (Ruifeng 125) × BB31	Resistance to Asian corn borer, armyworm and cotton bollworm	Ruifeng 125	Hangzhou Ruifeng Biotechnology Co., Ltd.; Zhejiang University
58	Nongda 778D	Corn	China Agricultural University	L239 (DBN9936) × C116A	Resistance to Asian corn borer, armyworm and cotton bollworm; glyphosate tolerance	DBN9936	Beijing Dabeinong Biotechnology Co., Ltd.
59	Liangyu 99D	Corn	Dandong Denghai Liangyu Seed Industry Co., Ltd.	LiangyuM03 (DBN9936) × LiangyuM5972	Resistance to Asian corn borer, armyworm and cotton bollworm; glyphosate tolerance	DBN9936	Beijing Dabeinong Biotechnology Co., Ltd.
60	Longping 218R	Corn	Anhui Longping High-Tech Seed Co., Ltd.	LB03 (Ruifeng 125) × LJ876	Resistance to Asian corn borer	Ruifeng 125	Hangzhou Ruifeng Biotechnology Co., Ltd.
61	Denghai 533K	Corn	Shandong Denghai Seed Industry Co., Ltd.	Denghai 22 × DH382 (ND207)	Resistance to Asian corn borer and cotton bollworm	ND207	Beijing Liangyuan Biotechnology Co., Ltd.
62	Denghai 685D	Corn	Shandong Denghai Seed Industry Co., Ltd.	DH382 (DBN9936) × DH357–14	Resistance to Asian corn borer, armyworm, cotton bollworm; glyphosate tolerance	DBN9936	Beijing Dabeinong Biotechnology Co., Ltd.
63	Denghai 7101	Corn	Shandong Denghai Seed Industry Co., Ltd.	DH382 (DBN9936) × DH357	Resistance to Asian corn borer, armyworm and cotton bollworm; glyphosate tolerance	DBN9936	Beijing Dabeinong Biotechnology Co., Ltd.
64	Huaxing Dan 88DT	Corn	Yunnan Shengyan Seed Industry Co., Ltd.	ZH08 (DBN3601T) × QR273	Resistance to Asian corn borer, armyworm and cotton bollworm; glyphosate and glufosinate tolerance	DBN3601T	Beijing Dabeinong Biotechnology Co., Ltd.
65	Zhonglian Dou 5046	Soybean	Institute of Crop Science, Chinese Academy of Agricultural Sciences	Fendou 99/Zhonghuang 6106	Glyphosate tolerance	Zhonghuang 6106	Institute of Crop Science, Chinese Academy of Agricultural Sciences
66	Zhonglian Dou 6024	Soybean	Institute of Crop Science, Hebei Academy of Agriculture and Forestry Sciences and Institute of Crop Science, Chinese Academy of Agricultural Sciences	Jidou 17//Heihe 38/Zhonghuang 6106	Glyphosate tolerance	Zhonghuang 6106	Institute of Crop Science, Chinese Academy of Agricultural Sciences
67	Maiyu 4003	Soybean	Beijing Dabeinong Biotechnology Co., Ltd.	Zhonghuang 35//Zhonghuang 13/DBN9004	Glyphosate and glufosinate tolerance	DBN9004	Beijing Dabeinong Biotechnology Co., Ltd.
68	Maiyu 526	Soybean	Beijing Dabeinong Biotechnology Co., Ltd.	Hefeng 50/DBN9004	Glyphosate and glufosinate tolerance	DBN9004	Beijing Dabeinong Biotechnology Co., Ltd.
69	Maiyu 503	Soybean	Beijing Dabeinong Biotechnology Co., Ltd.	Hefeng 50/DBN9004	Glyphosate and glufosinate tolerance	DBN9004	Beijing Dabeinong Biotechnology Co., Ltd.
70	Maiyu 511	Soybean	Beijing Dabeinong Biotechnology Co., Ltd.	Hefeng 50/DBN9004	Glyphosate and glufosinate tolerance	DBN9004	Beijing Dabeinong Biotechnology Co., Ltd.
71	Maiyu 579	Soybean	Beijing Dabeinong Biotechnology Co., Ltd.	Hefeng 50/DBN9004	Glyphosate and glufosinate tolerance	DBN9004	Beijing Dabeinong Biotechnology Co., Ltd.
72	Maiyu 565	Soybean	Beijing Dabeinong Biotechnology Co., Ltd.	Hefeng 50/DBN9004	Glyphosate and glufosinate tolerance	DBN9004	Beijing Dabeinong Biotechnology Co., Ltd.
73	Zhonglian Dou 1505	Soybean	Institute of Crop Science, Chinese Academy of Agricultural Sciences and Soybean Research Institute, Heilongjiang Academy of Agricultural Sciences	Heino 69//Habei 46–1/Zhonghuang 6106	Glyphosate tolerance	Zhonghuang 6106	Institute of Crop Science, Chinese Academy of Agricultural Sciences
74	Zhonglian Dou 1307	Soybean	Institute of Crop Science, Chinese Academy of Agricultural Sciences and Suihua Branch, Heilongjiang Academy of Agricultural Sciences	Beidou 40//Beidou 40//Heihe 38/Zhonghuang 6106	Glyphosate tolerance	Zhonghuang 6106	Institute of Crop Science, Chinese Academy of Agricultural Sciences
75	Zhonglian Dou 2825	Soybean	Institute of Crop Science, Chinese Academy of Agricultural Sciences and Hulunbuir Academy of Agricultural and Animal Science	Heihe 43//Heihe 43/Zhonghuang 6106	Glyphosate tolerance	Zhonghuang 6106	Institute of Crop Science, Chinese Academy of Agricultural Sciences
76	Zhonglian Dou 2109	Soybean	Hulunbuir Academy of Agricultural and Animal Science and Institute of Crop Science, Chinese Academy of Agricultural Sciences	Huajiang 2/Keshan 1/Zhonghuang 6106	Glyphosate tolerance	Zhonghuang 6106	Institute of Crop Science, Chinese Academy of Agricultural Sciences
77	Zhonglian Dou 2041	Soybean	Hulunbuir Academy of Agricultural and Animal Science and Institute of Crop Science, Chinese Academy of Agricultural Sciences	Huajiang 2/Kenfeng 20/Zhonghuang 6106	Glyphosate tolerance	Zhonghuang 6106	Institute of Crop Science, Chinese Academy of Agricultural Sciences
78	Zhonglian Dou 1309	Soybean	Suihua Branch, Heilongjiang Academy of Agricultural Sciences and Institute of Crop Science, Chinese Academy of Agricultural Sciences	Beidou 40//Beidou 40//Heihe 38/Zhonghuang 6106	Glyphosate tolerance	Zhonghuang 6106	Institute of Crop Science, Chinese Academy of Agricultural Sciences
79	Zhonglian Dou 1311	Soybean	Suihua Branch, Heilongjiang Academy of Agricultural Sciences, Institute of Crop Science, Chinese Academy of Agricultural Sciences and Soybean Research Institute, Heilongjiang Academy of Agricultural Sciences	Heino 69//Habei 46–1/Zhonghuang 6106	Glyphosate tolerance	Zhonghuang 6106	Institute of Crop Science, Chinese Academy of Agricultural Sciences
80	Zhonglian Dou 1510	Soybean	Institute of Crop Science, Heilongjiang Academy of Agricultural Sciences, Chinese Academy of Agricultural Sciences and Jilin Academy of Agricultural Sciences	Heinong 69//Habei 46–1/Zhonghuang 6106	Glyphosate tolerance	Zhonghuang 6106	Institute of Crop Science, Chinese Academy of Agricultural Sciences
81	Zhonglian Dou 1512	Soybean	Soybean Institute, Heilongjiang Academy of Agricultural Sciences and Institute of Crop Science, Chinese Academy of Agricultural Sciences	Heinong 69//Habei 46–1/Zhonghuang 6106	Glyphosate tolerance	Zhonghuang 6106	Institute of Crop Science, Chinese Academy of Agricultural Sciences

The symbols//and/in this table denote hybridization and backcrossing, respectively. For instance, in Table entry 66, Jidou 17//Heihe 38/Zhonghuang 6106 represents a genetically modified soybean variety that was developed by crossing the maternal parent, Jidou 17, with the paternal parent, a hybrid offspring that derived from Heihe 38, a non-transgenic soybean variety, and Zhonghuang 6106, a transgenic soybean variety. The resulting genetically modified variety, Jidou 17//Heihe 38/Zhonghuang 6106, has been designated as the Zhonglian Dou 6024 variety.

## 6. Commercialization of GM Food Crops in China

The commercialization of GMOs in China began in 1998 with the introduction of insect-resistant cotton that was not destined for processing as food or feed. In the early 21st century, China approved the commercial cultivation of GM tobacco, tomato, pepper, and poplar. In 2006, GM papaya became the first GM fruit to be approved for commercial planting. By 2021, the only GM plants cultivated commercially in China were insect-resistant cotton and virus-resistant papaya.^36^ In 2021, the area cultivated with GM crops in China was approximately 2.81 million hectares. Of this, GM cotton accounted for around 2.8 million hectares, representing roughly 95% of the country’s total cotton cultivation area. By contrast, the area planted with GM papaya was approximately 10,000 hectares.^37^ From 2021 onwards, the commercialization of GM staple crops entered a new phase, transitioning from gradual implementation to accelerated expansion, marking a shift toward widespread commercial application. In line with the established roadmap for GM biotechnology commercialization, China is currently advancing the commercial cultivation of GM corn and soybean that are intended for animal feed. To address agricultural challenges, such as the fall armyworm and weed resistance, China initiated field trials in 2021 for herbicide-tolerant soybean and insect-resistant, herbicide-tolerant corn, both of which had already received safety certifications. Initially, these trials were conducted in research test fields, covering relatively small pilot areas of approximately 76.67 hectares. By 2022, the commercial trials of GM corn and soybean had expanded beyond research sites to farmland, primarily held by large-scale farmers, in Inner Mongolia and Yunnan, which increased the cultivation area to approximately 5,333 hectares.^10^ In 2023, the scope of commercial trials further broadened, with GM corn and soybean being planted on approximately 266,667 hectares in 20 counties across five provinces: Hebei, Inner Mongolia, Jilin, Sichuan, and Yunnan.^10^ By 2024, the commercialization area had expanded to 7 provinces (or regions) and 47 counties (or cities). The GM varieties approved for commercial pilot cultivation included all insect-resistant and herbicide-tolerant corn, as well as herbicide-tolerant soybean varieties with independent intellectual property rights and demonstrated commercial potential. The total cultivation area reached approximately 667,000 hectares.^10^ However, given that China’s annual planting areas for maize and soybean are approximately 44 and 10 million hectares, respectively, this figure still reflects the relatively low adoption rates of GM maize and soybean. It highlights that China remains far from achieving the widespread commercialization of these GM crops. In the 3-year commercialization trials, insect-resistant corn demonstrated over 90% effectiveness in controlling pests, such as the fall armyworm, and both insect-resistant, herbicide-tolerant corn and herbicide-tolerant soybean yields showed average increases of 8.9% and 8.8%, respectively,^10^ reflecting substantial gains in productivity, efficiency, and ecological benefits. Between 2021 and 2024, during the phased commercialization trials of GM maize and soybean, China pioneered an innovative stepwise commercialization mechanism that facilitated the steady expansion of GM crop deployment. Notably, all GM maize and soybean varieties involved in these pilot programs had already obtained safety certificates. However, because the formal implementation of the national variety approval system had only begun at the end of 2023, these varieties had not yet completed the required approval procedures during the trial phase. According to the *Regulations on the Administration of Agricultural GMOs Safety*, GM varieties that have obtained safety certificates must still undergo national variety approval and obtain a seed production and marketing license before they can enter full commercial circulation. In the initial stage of the trials (2021), cultivation was strictly confined to designated research plots, subject to physical isolation standards and comprehensive oversight under the regulatory framework for GM experimental materials. As the trials expanded into large-scale farmer-managed fields between 2022 and 2024, regulatory authorities established a unified management model – centralized seed supply, coordinated procurement, and standardized technical protocols – to ensure full-chain oversight. The MARA also implemented regular inspection mechanisms to ensure the traceability of seed sources, and strictly prohibited seed saving by farmers. All the harvests from the trial program were required to be collected by designated enterprises. Furthermore, GM crops produced under this program were strictly limited to use in animal feed and industrial processing (e.g., oil extraction), and they were explicitly barred from entering the human food supply chain. Although the trial cultivation area reached 667,000 hectares by 2024, these large-scale trials were still classified as “commercial demonstration” rather than full commercialization, owing to the absence of completed variety approval and marketing license procedures. Importantly, the expansion in planting scale did not lead to any relaxation in regulatory oversight. All trial activities remain fully subject to the regulatory framework until the varieties pass national variety approval and receive seed production and distribution licenses, at which point regulatory restrictions may be lifted.

## 7. Challenges and Solutions in China’s GMO Safety Administration

Over the past 30 years, China has made significant advances in GMO research and development, the importation and processing of GM food crops, and the commercialization of insect-resistant cotton and domestically developed GM crops. As a result, China has established a regulatory model to ensure the safe development and commercialization of agricultural biotechnology. However, challenges have emerged, highlighting the need for the ongoing optimization and improvement of the regulatory framework.

In the legal and regulatory framework, there are potential conflicts between existing regulations. For instance, the *Administrative Rule for Genetic Engineering Safety*, issued by the MST, contradicts the *Regulation on the Safety Administration of Agricultural GMOs*, issued by the SC, because of differing implementing authorities. The former assigns responsibility to the MST, whereas the latter designates the MARA. However, the *Regulation on the Safety Administration of Agricultural GMOs* is considered superior to the *Administrative Rule for Genetic Engineering Safety* in the legal hierarchy. Additionally, current regulations fail to address all aspects of GMO safety management, leaving gaps in the framework. While the approval processes for intermediate trials, environmental releases, production trials, and safety certifications are well defined, there is insufficient regulatory coverage concerning laboratory research on GMOs. Both the *Regulation on the Safety Administration of Agricultural GMOs* and the *Administrative Rule for Safety Evaluation of Agricultural GMOs* merely state that research institutions can approve laboratory research for Level I and II GMOs independently, without oversight. This loophole has led to instances in which research institutions, after completing laboratory studies, bypassed legal approval and directly conducted intermediate trials, violating safety protocols.

Regarding imported GM food crops, current regulations require food and feed manufacturers to obtain production and processing licenses. Furthermore, imported GM crops are restricted to use as food or feed ingredients and cannot be released into the environment. However, with the commercialization of domestically grown GM corn and soybean, the question of whether harvested GM crops also require administrative licenses for food and feed processing has arisen. Moreover, the regulation of commercially planted GM food crops remains ambiguous. If regulation is necessary, then what form should it take? Because soybean originated in China, the commercialization of GM soybean raises concerns about protecting wild soybean resources from gene flow.^38,39^ Therefore, China should strengthen its regulatory framework to align with technological advancements, preventing delays in the responsible application and development of GMOs.

Regarding information disclosure and public participation, China’s GMO safety management has been marked by a lack of proactivity and timely communication. Prior to 2010, public attitudes toward GMOs were generally positive. However, after 2010, a lack of proactive communication, coupled with negative publicity surrounding GMO food safety, led to a shift in public perception.^40^ The shift in the Chinese public’s attitude toward the application of GM technology around 2010 resulted from the complex interplay of multiple factors, including policy direction, media environment, public psychology, and international context. Prior to 2010, public perceptions of GM biotechnology research and its commercial applications were generally positive. This optimism was largely driven by strong policy support, continuous technological progress, insights gained from international experience, and increasing market demand. Additionally, the relatively homogeneous media environment during the early stages helped reinforce a positive narrative. In 2008, the Chinese government approved the establishment of the *Major Special Project for the Development of New GM Varieties*, signaling strong support for GM research and innovation. In 2009, the government officially proposed the commercialization of GM technology, conveying confidence in its future prospects. The successful adoption of insect-resistant cotton further strengthened public trust in the practical benefits of GM technologies. Additionally, large-scale GM crop cultivation in countries such as the United States offered valuable international reference points, and the fact that imported GM maize and soybean were used exclusively for feed and industrial processing, rather than for direct human consumption, helped avoid triggering strong public concern. More importantly, prior to 2010, the limited penetration of digital technologies, such as the internet and smartphones, meant that GMO safety had yet to emerge as a mainstream public issue. Public understanding of GM technology was largely shaped by the official discourse from the scientific and policy communities, and negative controversies surrounding GMOs had not yet been amplified in the public sphere. After 2010, however, the rapid proliferation of internet access, smartphones, and social media platforms ushered in an era of “self-media” in China, in which rumors and negative narratives about GM technology could circulate widely and rapidly. In this context, negative information often spreads with greater intensity and reach. At the same time, public science communication efforts related to GMO safety remained limited, and the government failed to respond promptly to counter misinformation. This lack of timely engagement was a key factor contributing to the post-2010 shift in public sentiment toward GM technology. Ultimately, this transformation in public attitude reflects a complex outcome shaped by evolving perceptions of technological risk, the transparency of policy processes, shifting social dynamics, and changing international influences. Although the Chinese government has since worked to strengthen regulatory oversight and expand science communication efforts, the delayed response and accumulation of negative public discourse have resulted in a growing “trust deficit.” Additionally, mechanisms for public participation in GMO safety management remain underdeveloped, and the regulatory framework lacks sufficient channels for public input, which has contributed to a lack of public trust in regulatory decision-making. To address these issues, China could learn from international best practices, such as the EU’s regulation on food risk assessment transparency^41,42^ and Japan’s disclosure standards for gene-edited organisms,^43^ to enhance transparency, improve public confidence, and refine GMO safety management.

In terms of commercial release management, China’s regulatory process remains complex and burdensome. Obtaining an agricultural GMO safety certificate typically takes 7–12 years, which significantly delays commercialization. For example, the evaluation criteria for insect resistance and herbicide tolerance required in the application process for GMO safety certificates overlap with those required in the variety approval process. However, the two systems do not allow for data sharing. Under the current regulatory framework, developers must first obtain a safety certificate before applying for variety approval. However, during the variety approval stage, they are required to repeat evaluations of insect resistance, herbicide tolerance, and agronomic traits. These redundancies increase both the time and financial costs for developers and, to some extent, delay the commercialization of GM crops. Additionally, GMO variety approval is currently only available at the national level, whereas conventional crops can be approved at the provincial level. To accelerate the approval timeline without compromising safety, China should streamline the safety evaluation process and implement clearer, more efficient evaluation procedures.

In terms of management capacity, China has established a relatively comprehensive legal and regulatory framework; however, challenges remain in the actual supervision of GMOs. Regulatory personnel at various levels, particularly those at the provincial and lower levels, lack expertise and specialized skills in GMO safety management. For example, the unauthorized field trials and subsequent illegal dissemination of GM rice in Hubei Province, a central region of China, during 2004–2005 exemplified a significant lapse in local biosafety oversight. The incident stemmed from weak regulatory control over GM experimental materials by supervisory authorities, coupled with inadequate containment measures implemented by researchers. Consequently, some local farmers gained illicit access to these experimental materials during the trial period. Driven by economic incentives, particularly the reduction in pesticide costs afforded by insect-resistant GM rice, certain farmers took the risk of planting the unauthorized seeds. Although penalties were eventually imposed on the researchers, seed companies, and farmers involved, enforcement efforts primarily targeted downstream actors and failed to address the upstream sources of dissemination. This case revealed several structural deficiencies in local biosafety governance. Notably, China’s predominantly household-based, highly decentralized smallholder farming system poses major challenges for comprehensive regulatory enforcement. In such a fragmented agricultural landscape, it is exceedingly difficult for local authorities to monitor rice production effectively at the village or household level. Furthermore, detecting GM content necessitates specialized laboratory equipment, which is typically unavailable at the grassroots level. As a result, local regulatory agencies often lack the technical capacity to conduct frequent and wide-range sampling across their jurisdictions. Furthermore, local regulatory personnel are generally unfamiliar with relevant regulations and policies. According to existing laws, agricultural authorities oversee GMOs and their products before market entry, whereas market regulatory authorities manage products once they are on the market. In cases involving food safety concerns, health authorities are also engaged. Therefore, the safety management of GMOs and related products necessitates multi-departmental coordination, which poses significant challenges in practice. For example, imported GM soybean has been released into the environment and used improperly.^44–46^ The illegal release of imported GM soybean into the environment or their diversion from approved uses is primarily driven by profit-seeking behaviors of individuals and enterprises that deliberately violate regulations for economic gain. Because the price of domestically produced non-GM soybean is generally higher than that of imported GM soybean, some violators are willing to take legal risks by misrepresenting imported GM soybean as domestic non-GM varieties to exploit the price differential for additional profit. According to the administrative division of responsibilities in China, the regulation of products entering market circulation falls under the jurisdiction of the market supervision administration, whereas the oversight of agricultural GMOs is managed by agricultural authorities. Therefore, the effective regulation of the illegal diversion and environmental release of imported GM soybean requires close coordination between market supervision agencies and agricultural regulatory bodies. To address this successfully, it is essential not only to enhance training for regulatory personnel on GMO safety, policies, and regulations, but also to improve interagency communication and coordination in management and enforcement. The interagency coordination mechanism (an inter-ministerial joint meeting mechanism, in which relevant ministries collaboratively discuss and coordinate on major issues in GMO safety management that require cross-agency cooperation) should be strengthened with clear responsibility delineation to create an efficient communication system that boosts regulatory effectiveness. Additionally, increased funding and a stronger regulatory capacity are critical. Agricultural administrative departments below the provincial level currently face insufficient support in terms of personnel, funding, and institutional resources, which limits their ability to effectively manage GMO safety. Local regulatory agencies often struggle with complex GMO safety issues, hindering effective oversight. Regulatory personnel must possess specialized knowledge and skills to manage these challenges; however, there are significant gaps in both expertise and technological resources.

Regarding target pest resistance management, current measures primarily focus on revising regulations and policies that impede the acceleration of GMO industrialization. However, insufficient attention has been given to managing resistance in target pests following the widespread use of GMOs. In the promotion and utilization of GM food crops, ongoing resistance monitoring and management tailored to regional conditions, along with the implementation of strategies suited to China’s unique circumstances, are imperative. Therefore, regulations and policies for the sustainable use of GMO technology should be developed, including continuous, real-time resistance monitoring and management in large-scale commercial planting areas. The timely implementation of resistance management strategies is also necessary. The complexity and diversity of GMO technologies increase the difficulty of balancing commercialization with ecological preservation in technical evaluations. A major challenge lies in fostering the development of GMO technology while maintaining both biosafety and an ecological balance. Achieving this balance requires coordinated efforts across science, technology, policy, public education, and other sectors. To ensure that GMO commercialization progresses without compromising ecological sustainability, it is essential to establish a comprehensive framework that integrates rigorous scientific assessments, evidence-based policy, and regulatory measures, along with environmentally responsible agricultural practices, active public engagement, and strengthened international collaboration. These elements, when effectively coordinated, can help mitigate ecological risks and support the safe and sustainable deployment of GMO technologies. In the global context, GMO safety governance has emerged as both a strategic frontier for international cooperation and a focal point of regulatory competition among countries, reflecting the increasing importance of harmonized standards, risk communication, and transboundary oversight in the biotechnology sector. China must actively engage in international cooperation, drawing from advanced international experiences and technologies while addressing competition and pressures from other countries. As part of sharing China’s GMO safety management model with the world, China should participate in international organizations, such as the Food and Agriculture Organization, the World Health Organization, and the Codex Alimentarius Commission, contribute to international standard-setting, promote global coordinated management, and enhance the global capacity to address ecological challenges.^47–49^

## 8. Conclusion

Compared with the well-established agricultural biotechnology research and commercialization system in the United States, China’s efforts in GMO research and application are relatively recent, leading to a noticeable gap, particularly in the discovery and utilization of key functional genes, the development of GMOs, and the rate of commercial adoption. Additionally, the pace of GM staple crop commercialization in China has not yet met the expectations set by the Chinese government for accelerating the commercialization of crops produced by biotechnology breeding.

In GMO safety management, China has consistently adopted a cautious approach, drawing on international practices, focusing on both products and processes, and striving to ensure the safety of GMOs while advancing research and application. The design of China’s regulatory framework reflects policies tailored to national conditions that also align with international standards and safeguard national interests. Consistent with international organizations and the practices of most countries, China has enacted comprehensive regulations covering all aspects of GMO safety management, including research, testing, production, processing, and trade. These regulations, such as the *Regulation on the Safety Administration of Agricultural GMOs* and supporting rules, provide a robust legal framework for the continued development of China’s GMO industry. Although the system is characterized by a rigorous regulatory design, a solid evaluation framework, and strong technical support, challenges remain, including regulatory conflicts, gaps in post-commercialization oversight, mismatches between labeling policies and commercial progress, and low levels of public engagement in GMO safety governance in China. Nevertheless, by balancing the need to ensure human, animal, and environmental safety with the goal of fully harnessing the benefits of GMOs, China has developed a distinctive approach to GMO safety management. This approach is characterized by a tiered trial system, a stepwise approval process, and comprehensive, whole-process regulatory oversight that is specifically designed to align with China’s agricultural production structure and national conditions, thereby offering valuable insights to the international community.

## Supplementary Material

Supplemental Material

Supplemental Material

## Data Availability

The authors have nothing to report.
