# Correction

**DOI:** 10.1080/21645698.2025.2524997

**Published:** 2025-06-30

**Article title**: Initiating the commercialization of genetically modified staple crops in China:

domestic biotechnological advancements, regulatory milestones, and governance frameworks

**Authors**: Mou, T., Song, Q., Liu, Y., and Song, J.

**Journal**: *GM Crops & Food*

**DOI**: https://doi.org/10.1080/21645698.2025.2520664

The above article was initially published online with a missing information in the reference list. It has now been republished with the necessary addition, detailed below:

In the reference list, the 25th reference has been updated as follows:

Chen SY. 2023. Research on legal and regulatory issues for the commercial cultivation of transgenic crops [master’s thesis]. Fuzhou, China: Fujian Agriculture and Forestry University. doi: 10.27018/d.cnki.gfjnu.2023.000846.

