# Supplementary material for: Initiating the commercialization of genetically modified staple crops in China: domestic biotechnological advancements, regulatory milestones, and governance frameworks
Source: GM Crops Food. 2025 Jun 20;16(1):450–81. doi: 10.1080/21645698.2025.2520664 (PMC12218729; doi:10.1080/21645698.2025.2520664)
Supplement: Supplemental Material [file KGMC_A_2520664_SM6660.docx]

Supplementary Table 1. Overview of agricultural technology development and biotechnology-related breeding policies in China’s No. 1 document (1982–2025)

| Year | Policies on corn and soybean industry development, which are highly dependent on international markets | Agricultural technology development and biotechnology-related breeding policies |
| --- | --- | --- |
| 1982 |  | Focused on aligning the agricultural, forestry, livestock, and fishery industries for both short-term and long-term production needs. Key research projects, including the breeding of superior varieties and targeted technological advancements, were prioritized. |
| 1983 |  | Continued selection of critical research projects impacting production, such as superior variety breeding, pest and disease control, plant and animal quarantine, and the comprehensive utilization of biological resources. Breakthroughs and systematic scientific data accumulation were emphasized. |
| 1984 |  | Agricultural technology-related issues were not mentioned. |
| 1985 |  | Agricultural technology-related issues were not mentioned. |
| 1986 |  | Increased reliance on scientific research along with greater support to enhance crop yield per unit area. |
| 2004 |  | Strengthened programs for breeding superior grain crop varieties and for pest control. Priority was given to promoting influential, high-quality varieties and advanced technologies in key production regions. |
| 2005 |  | Emphasized basic agricultural research and the development of key technologies, which accelerated advancements in biotechnology and other high-tech fields. |
| 2006 |  | Focused on high-tech research in agriculture and continued the implementation of agricultural technology-related commercialization projects. Efforts were aimed at achieving significant agricultural technological breakthroughs with independent intellectual property. |
| 2007 |  | Launched special research initiatives for the agricultural sector that supported technology projects. Enforced labeling regulations for GM foods and other agricultural products. |
| 2008 |  | Initiated a major project on GM crop breeding, accelerating the implementation of seed and livestock improvement programs. |
| 2009 |  | Continued advancing GM crop breeding, integrating research resources and intensifying development efforts towards the production of GM varieties with enhanced pest and disease resistance, enhanced stress tolerance, increased yield, and improved quality, while promoting industrialization. |
| 2010 |  | Accelerated the innovation and application system for agricultural biotechnology. Continued implementing major GM crop breeding projects, focusing on the application of valuable functional genes and development of GM varieties with independent intellectual property, as well as promoting their industrialization under the framework for scientific evaluation and legal management. |
| 2011 |  | Agricultural technology-related issues were not mentioned. |
| 2012 | Expanded the production of key varieties, focusing on improving yield and quality. Supported the construction of production bases for cotton, oilseeds, and sugar in advantaged production areas. | Significant advancements were made in basic agricultural research, particularly in the genetic regulation and molecular breeding of agricultural organisms. Focused on plant and animal resistance mechanisms, pest control, and biosafety. Accelerated research on emerging technologies in agricultural biotechnology and precision agriculture, leading to major innovations with independent intellectual property rights, which positioned China at the forefront of modern agricultural technology. |
| 2013 | Focused on increasing crop yields to ensure abundant harvests. | Agricultural technology-related issues were not mentioned. |
| 2014 |  | Strengthened basic research and biotechnology development, with a focus on crop molecular breeding. |
| 2015 |  | Accelerated agricultural technology innovation, achieving breakthroughs in agriculture-related biotechnology and breeding. |
| 2016 |  | Implemented key projects in agricultural technology innovation, emphasizing breakthroughs in biotechnological breeding. Strengthened research in, and the regulation of, agricultural GM technologies, ensuring safety prior to cautious promotion. |
| 2017 | Increased the production of high-quality edible soybean, tubers, and miscellaneous grains and beans. | Strengthened efforts to promote independent innovation in the seed industry, alongside collaborative breeding programs for key crops. Expedited the development of high-quality, high-yield, multi-resistant, and widely adaptable crop varieties. |
| 2018 |  | Agricultural technology-related issues were not mentioned. |
| 2019 | Implemented the Soybean Revitalization Plan, which expanded planting areas using multiple strategies. Supported oilseed production in the Yangtze River Basin and reinforced the capacity for cotton, oilseed, sugar, and natural rubber production. | Launched a strategic action plan targeting critical agricultural technologies , which fostered innovation in agricultural technology. Emphasized efforts to promote self-innovation in biotechnology and seed industries. |
| 2020 | Increased support for high-yield soybean varieties and promoted new agricultural practices, such as intercropping soybeans and corn. | Agricultural technology-related issues were not mentioned. |
| 2021 | Improved subsidy policies for corn and soybean producers to stabilize soybean production, while promoting oilseed and peanut development through various measures. | Provided long-term, stable support for foundational breeding research and key breeding projects. Major agricultural biotechnology breeding programs were accelerated, with a focus on scientific integrity and strict rules to ensure the orderly use of biotechnology applications in industry. |
| 2022 | Actively implemented projects to enhance soybean and oilseed production capacities. | Launched major agricultural biotechnology breeding initiatives. |
| 2023 | Expanded soybean and oilseed plantings. Advanced the Soybean and Oilseed Production Capacity Enhancement Project. Supported strip planting of soybean and corn, as well as rotation systems, in the Northeast and Huang-Huai-Hai regions. Promoted the development of saline-alkali-tolerant soybean for cultivation. | Biotechnology breeding initiatives were comprehensively implemented, with efforts focused on advancing national breeding collaborations and livestock genetic improvement programs. Accelerated the development of new varieties, including high-yield, high-oil soybean, short-maturity canola, and salt-tolerant crops. Advanced the industrialization of biotechnological corn and soybean breeding, with the systematic expansion of pilot programs and standardized management practices. |
| 2024 | Consolidated achievements in expanding soybean plantings, supported the development of high-oil, high-yield varieties, and continued implementing soil fertility protection measures and subsidies for corn and soybean producers. | Intensified efforts to expand and accelerate the industrialization of biotechnological crop breeding. |
