# Supplementary material for: Initiating the commercialization of genetically modified staple crops in China: domestic biotechnological advancements, regulatory milestones, and governance frameworks
Source: GM Crops Food. 2025 Jun 20;16(1):450–81. doi: 10.1080/21645698.2025.2520664 (PMC12218729; doi:10.1080/21645698.2025.2520664)
Supplement: Supplemental Material [file KGMC_A_2520664_SM6471.docx]

Supplementary Table 2. Key policies related to agricultural biotechnology development in nine of China’s Five-Year Plans (1981–2025)

| Time Period | Five-Year Plan | Key Policies on Agricultural Biotechnology Development |
| --- | --- | --- |
| 1981–1985 | 6th Five-Year Plan | Over 30 new wheat varieties were developed, with experimental cultivation spanning 40 million acres, accounting for 10% of the national wheat acreage and resulting in a 5%–10% yield increase. In total, 40 new rice varieties were developed and planted across 50 million acres, resulting in an average approximate 50 kg per acre yield increase. In addition, 46 new vegetable varieties were developed and widely disseminated. Virus-free potato seedling technology was refined to address issues of degeneration and yield loss caused by viral infection, resulting in a 50%–100% increase in yield per acre. This breakthrough resolved key technical challenges in national seed potato production. Additionally, four high-quality hybrid combinations and five fast-growing hybrid combinations of yellow-feathered broiler chickens were selected. These chickens gained 50% more weight and used 33% less feed than local breeds. |
| 1986–1990 | 7th Five-Year Plan | Improved crop varieties were developed and the practical application of scientific advancements encouraged. Significant progress was made in updating varieties of major crops such as grains, cotton, and oilseeds, improving quality, resistance, and productivity by 10%. A total of 397 new varieties across 14 major crops, including rice, wheat, maize, cotton, soybean, and rapeseed, were developed, with a cumulative planting area of 640 million acres, which increased grain and cotton yields to 14 billion kg and 3.3 billion kg, respectively. |
| 1991–1995 | 8th Five-Year Plan | Increasing grain yields was the focus. The development of 473 new varieties of grains, economic crops, and vegetables resulted in an average yield increase of over 10%. New varieties of rice, wheat, and maize were planted across 1.28 billion acres, contributing to an additional 30 billion kg of grain. |
| 1996–2000 | 9th Five-Year Plan | Ensuring a stable supply of agricultural products, increasing farmers' incomes, improving the agricultural ecological environment, and accelerating agricultural industrialization were emphasized. In total, 22 major projects and over 800 sub-projects were launched. A total of 664 new crop varieties were developed, 988 new products were introduced, and 1,995 experimental bases and 4,807 demonstration sites were established. The development and testing of high-yield integrated technologies for major crops such as wheat and rice led to the planting of 4.5 billion acres, which produced 150 billion kg of grain. |
| 2001–2005 | 10th Five-Year Plan | Major projects on functional genomics and biotechnology were initiated. China led international efforts by sequencing the complete rice genome (subspecies 9311). The research focused on using agricultural genomics to improve crops, protect and use biological resources more efficiently, and make farming environments and resources more environmentally friendly. This period laid the foundation for modern agricultural technology and encouraged original innovations in agricultural science and technology. |
| 2006–2010 | 11th Five-Year Plan | The development of GM crop varieties was the focus. Core technologies, including gene cloning and validation, large-scale genetic modification, and biosafety evaluation, were advanced. Three technological platforms were established to improve germplasm innovation, create new varieties, and produce various seeds. Over 1,000 new functional genes were discovered. The GM breeding system was further developed, leading to the creation of 100–150 GM crop varieties and over 30 GM animal breeds/materials. |
| 2011–2015 | 12th Five-Year Plan | The implementation of major GM crop development projects was continued. Key breakthroughs were achieved in gene cloning, functional validation, large-scale genetic modification, and biosafety. Efforts focused on developing new GM varieties of cotton, maize, and other major crops, as well as improving resistance to pests, diseases, and environmental stresses. These advances contributed to the industrialization of new GM varieties, enhancing China’s agricultural biotechnology innovation capacity. |
| 2016–2020 | 13th Five-Year Plan | Efforts to overcome key technological challenges in GM crop development were continued. Research into genes conferring insect resistance, disease resistance, drought tolerance, and cold tolerance was intensified. The commercialization of major GM products, such as insect-resistant cotton, maize, and herbicide-tolerant soybean, was accelerated. New technologies for gene cloning, genetic modification, and biosafety evaluation were developed, with a focus on improving traits in staple crops, such as rice and wheat, using techniques such as non-endosperm specific expression and gene editing. A standardized biosafety evaluation system was established to ensure the safety of GM products. |
| 2021–2025 | 14th Five-Year Plan | Innovations in biotechnology breeding systems and the development of breakthrough new varieties are the foci. Bottlenecks in the efficient genetic transformation of major agricultural plants and animals are being targeted. New technologies for genetic transformation that do not rely on receptor genotypes or multi-gene stacking are being developed. Agricultural biotechnology data, including genomics, metabolomics, and phenomics, are being integrated to create new technologies for polyploid breeding, rapid domestication, and cross-species improvement. The plan emphasizes a science-based approach, strict regulation, and the orderly industrialization of biotechnology. |
